# Physical Fitness Training in Patients with Subacute Stroke (PHYS-STROKE): multicentre, randomised controlled, endpoint blinded trial

**DOI:** 10.1136/bmj.l5101

**Published:** 2019-09-18

**Authors:** Alexander H Nave, Torsten Rackoll, Ulrike Grittner, Holger Bläsing, Anna Gorsler, Darius G Nabavi, Heinrich J Audebert, Fabian Klostermann, Ursula Müller-Werdan, Elisabeth Steinhagen-Thiessen, Andreas Meisel, Matthias Endres, Stefan Hesse, Martin Ebinger, Agnes Flöel

**Affiliations:** 1 Centre for Stroke Research Berlin, Charité–Universitätsmedizin Berlin, Berlin, Germany; 2 Klinik und Hochschulambulanz für Neurologie, Charité–Universitätsmedizin Berlin, Berlin, Germany; 3 German Center for Cardiovascular Research, partner site Berlin, Germany; 4 Berlin Institute of Health, Berlin, Germany; 5 Kliniken Beelitz, Beelitz-Heilstätten, Germany; 6 Institute of Biometry and Clinical Epidemiology, Charité–Universitätsmedizin Berlin, Berlin, Germany; 7 Median Klinik Grünheide, Grünheide, Germany; 8 Vivantes Klinikum Neukölln, Klinik für Neurologie, Berlin, Germany; 9 Evangelisches Geriatriezentrum Berlin, Berlin, Germany; 10 NeuroCure Clinical Research Center, Charité–Universitätsmedizin Berlin, Berlin, Germany; 11 German Center for Neurodegenerative Diseases, partner site Berlin, Germany; 12 Medical Park Berlin Humboldtmühle, Berlin, Germany; 13 Department of Neurology, University Medicine Greifswald, Ferdinand-Sauerbruch-Strasse, 17475 Greifswald, Germany; 14 German Center for Neurodegenerative Diseases, partner site Rostock/Greifswald, Germany

## Abstract

**Objective:**

To determine the safety and efficacy of aerobic exercise on activities of daily living in the subacute phase after stroke.

**Design:**

Multicentre, randomised controlled, endpoint blinded trial.

**Setting:**

Seven inpatient rehabilitation sites in Germany (2013-17).

**Participants:**

200 adults with subacute stroke (days 5-45 after stroke) with a median National Institutes of Health stroke scale (NIHSS, range 0-42 points, higher values indicating more severe strokes) score of 8 (interquartile range 5-12) were randomly assigned (1:1) to aerobic physical fitness training (n=105) or relaxation sessions (n=95, control group) in addition to standard care.

**Intervention:**

Participants received either aerobic, bodyweight supported, treadmill based physical fitness training or relaxation sessions, each for 25 minutes, five times weekly for four weeks, in addition to standard rehabilitation therapy. Investigators and endpoint assessors were masked to treatment assignment.

**Main outcome measures:**

The primary outcomes were change in maximal walking speed (m/s) in the 10 m walking test and change in Barthel index scores (range 0-100 points, higher scores indicating less disability) three months after stroke compared with baseline. Safety outcomes were recurrent cardiovascular events, including stroke, hospital readmissions, and death within three months after stroke. Efficacy was tested with analysis of covariance for each primary outcome in the full analysis set. Multiple imputation was used to account for missing values.

**Results:**

Compared with relaxation, aerobic physical fitness training did not result in a significantly higher mean change in maximal walking speed (adjusted treatment effect 0.1 m/s (95% confidence interval 0.0 to 0.2 m/s), P=0.23) or mean change in Barthel index score (0 (−5 to 5), P=0.99) at three months after stroke. A higher rate of serious adverse events was observed in the aerobic group compared with relaxation group (incidence rate ratio 1.81, 95% confidence interval 0.97 to 3.36).

**Conclusions:**

Among moderately to severely affected adults with subacute stroke, aerobic bodyweight supported, treadmill based physical fitness training was not superior to relaxation sessions for maximal walking speed and Barthel index score but did suggest higher rates of adverse events. These results do not appear to support the use of aerobic bodyweight supported fitness training in people with subacute stroke to improve activities of daily living or maximal walking speed and should be considered in future guidelines.

**Trial registration:**

ClinicalTrials.gov NCT01953549.

**Figure:**
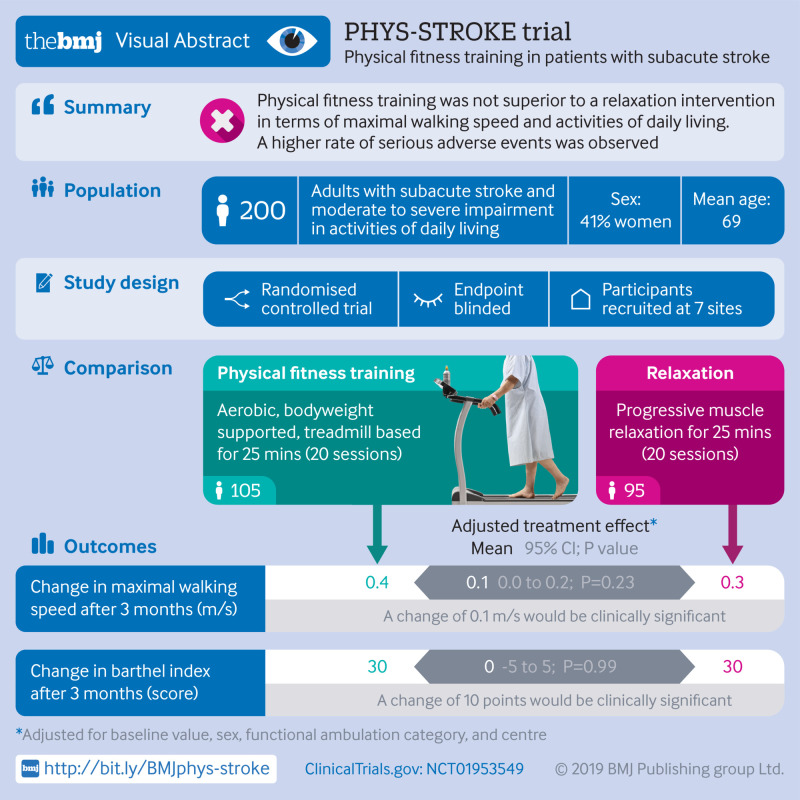


## Introduction

Despite encouraging advances in the early treatment of stroke,^1^ at least one third of the 10 million people worldwide with new stroke each year^2^ remain functionally dependent and as a result experience impairments in activities of daily living.^3^ ^4^ The number of stroke survivors with impairments in activities of daily living is increasing, leading to more people with stroke who are dependent on rehabilitation interventions.^5^ To date, no drug treatments are available to enhance rehabilitation. Treadmill based physical fitness training constitutes a non-drug approach in stroke rehabilitation that might not only prevent deconditioning but also show associated benefits on activities of daily living, such as walking and climbing stairs.^6^ ^7^ ^8^

A meta-analysis of small randomised controlled trials showed improvements in speed and tolerance of walking after physical fitness training in stroke survivors.^6^ The studies have, however, varied in type and intensity of exercise, timing of initiation after stroke, and control groups.^7^ ^9^ ^10^ ^11^ The American Heart Association/American Stroke Association currently recommends aerobic exercise for stroke survivors, with three to five sessions weekly lasting 20 to 60 minutes and at a heart rate of 55-80% of the maximum. Applied in the subacute stage after stroke, aerobic exercise is thought to promote neuroplasticity and to have beneficial effects on functional outcomes.^7^ So far, nine randomised controlled trials (n=324) have compared the effects of aerobic physical fitness training on maximal walking speed—an important indicator of mobility in everyday life^6^—with a control intervention. However, only two of these studies (n=73) enrolled participants within the first six weeks after stroke, and these participants showed improvement in maximal walking speed post-intervention.^6^ For activities of daily living and disability, two small randomised rehabilitation trials (n=199) that applied 400-540 minutes of physical fitness training in the early and late subacute stage after stroke found an increase in the Barthel index score, a disability scale widely used in the clinical setting to measure activities of daily living.^12^ ^13^ Meta-analyses have, however, indicated that the evidence for improvement in Barthel index scores after physical fitness training is still inconclusive.^6^

Previously, the larger Locomotor Experience Applied Post-Stroke (LEAPS) trial randomised 408 participants to treadmill based locomotor training either two or six months after stroke, or to a progressive exercise programme at home, and did not detect a difference in treatment effects.^11^ LEAPS did not, however, apply an aerobic physical fitness training early after stroke.

We performed a multicentre, randomised controlled trial in adults with stroke in the early subacute phase (days 5-45 after stroke) to determine the efficacy of aerobic treadmill based, physical fitness training on maximal walking speed and activities of daily living compared with relaxation as a control intervention.

## Methods

### Study design

The study protocol for the multicentre, randomised controlled, endpoint blinded Physical Fitness Training in Patients with Subacute Stroke (PHYS-STROKE) trial is available online (https://doi.org/10.1186/1745-6215-15-45) and has been published previously.^14^ All participants provided informed consent. Adults were enrolled at seven inpatient rehabilitation sites in Berlin, Germany, and the surrounding area. A medical monitoring and independent data safety and monitoring board was appointed by the Centre for Stroke Research Berlin. Guidance of study centre representatives was maintained by regular telephone contact and study visits by members of the coordinating trial centre at the Centre for Stroke Research Berlin. To determine eligibility for the trial, trained trial physicians screened people with an imaging confirmed diagnosis of ischaemic or haemorrhagic stroke who had been admitted to hospital within a recruiting centre.

### Participants

People were eligible for the trial if they were aged at least 18 years, were in the subacute phase of ischaemic or haemorrhagic stroke (days 5-45 after stroke onset), were able to sit unsupported for at least 30 seconds, were considered able to perform aerobic exercise by the responsible trial physician, and had a Barthel index score of 65 or less at the time of enrolment. The Barthel index measures activities of daily living based on 10 items, with scores ranging from 0 to 100 points—higher scores indicating less dependence.^15^ Key exclusion criteria were intracranial haemorrhage from a ruptured aneurysm or arteriovenous malformation, inability to perform required physical exercise, assisted walking before stroke, or severe cardiac or psychiatric comorbidities. If study requirements were met, trial physicians assessed information on stroke type and medical conditions after written informed consent was obtained. The supplementary file lists the inclusion and exclusion criteria.

### Randomisation and blinding

Participants were stratified by age (≤65 years, >65 years), functional ambulation category (scores ≤3, >3), and centre and were randomised using a web based tool in a 1:1 ratio to receive aerobic physical fitness training or relaxation sessions (control group) in addition to standard care.^16^ The functional ambulation category is a six point scale that assesses dependency on walking aids, with higher scores indicating less dependency. Trained study assessors (physiotherapists, occupational therapists) collected information on participant characteristics and clinical data from chart reviews at baseline visits and carried out outcome assessments at baseline and follow-up. Study assessors and the trial statistician were blinded to the intervention allocation.

### Interventions

Therapists at each rehabilitation site performed interventions according to standardised protocols taught by a manual and two day training course. The study interventions were applied during inpatient stay at the rehabilitation centre, in addition to standard rehabilitation therapy according to German guidelines (www.bar-frankfurt.de). The supplementary file provides detailed information on the amount and content of standard care. Each study session was for 50 minutes (therapist led), comprising 25 minutes of core intervention (training aimed at target heart rate or relaxation time), and took place five times weekly over four weeks (20 sessions in total). The start and end of each intervention session as well as the duration of the core intervention were documented for each participant. An intervention period of four weeks was chosen to ensure intervention sessions were applied during the length of the inpatient stay at the rehabilitation centres. This also enabled the assessment of short term outcomes at three months after stroke.

The supplementary appendix provides a detailed description of the aerobic physical fitness training and relaxation interventions. Briefly, the aerobic physical fitness training sessions included treadmill based, bodyweight supported training at a cardiorespiratory active (aerobic) level to reach a target heart rate for 25 minutes. The target heart rate was calculated by the formula 180 minus years of age, a pragmatic decision, which resembles conventional approaches for calculation of target heart rate that is 50-60% of each participant’s maximum heart rate (see supplementary appendix). If participants used β blockers, we reduced the target heart rate by 10 beats per minute. Participants used a bodyweight supported treadmill (Multi-disk treadmill Callis, Model Therapie; Sprintex Trainingsgeräte, Kleines Wiesental, Germany; Reha-Stim, Berlin, Germany) if their functional ambulation category score was 3-5 or an electromechanical gait trainer (Gait Trainer GT1; Reha-Stim, Berlin, Germany) if their score was 0-2. The amount of bodyweight support was applied as required. If necessary, one or two therapists assisted with leg movement, such as extending hip and knee, shifting body weight, or setting the paretic leg in the case of severe paresis of the peroneus muscles. Participants used the same orthoses during the intervention as during standard care physiotherapy. Each training session, including preparation time and a warm-up and cool-down phase, lasted 50 minutes and comprised 25 minutes of active training at aimed target heart rate, depending on each participant’s ability, as recommended in current guidelines.^7^ To ensure target heart rate was maintained, heart rate during training was controlled through a pulse sensor (Polar FT1 HRM, Polar Electro Oy, Kempele, Finland) and a screen attached to the treadmill or gait trainer. Reduction of bodyweight support and an increase of belt speed or increase of inclination, or both was used to reach the target heart rate throughout the four week intervention, and thus to constantly induce aerobic training effects. The trainers documented changes of these variables during the intervention period and individual perceived exertion after each training session in intervention diaries. To prevent falls, participants were equipped with a modified parachute harness (Belt system; Reha-Stim, Berlin, Germany).

Relaxation sessions were performed as an active control and focused on contraction and relaxation of muscle groups in the face, arms, shoulders, back, and abdomen for 25 minutes. Participants were instructed to contract the muscles for five to 10 seconds then to relax for 30-40 seconds and were encouraged to pay attention to the feelings of warmth and heaviness. Sessions aimed to promote mental and physical relaxation and avoid any cardiovascular stress. Participants’ heart rates were monitored during the relaxation sessions, and ratings of perceived exertion were assessed at the end of each session. Participants in both groups received individual attention during each session to achieve comparability.

No specific treatment policy was prescribed after the intervention period. If participants stopped the intervention prematurely, we continued clinical follow-up at set time points. Participants were analysed in the per protocol analysis if they received 75% or more of the scheduled intervention sessions, had not suspended the intervention for more than five consecutive days, and had participated in the follow-up visit three months after stroke.

### Outcome measures

Study visits for outcome assessment were performed before and after the intervention period as well as three and six months after stroke. The primary outcome measures were change in maximal walking speed (assessed in m/s) and change in Barthel index scores three months after stroke compared with baseline in the intention-to-treat analysis. Maximal walking speed was assessed in a 10 m walk test.^17^ Participants were asked to walk at maximal speed for 14 m—2 m for acceleration and 2 m for deceleration, with markings on the floor for starting point, at 2 m (start of measurement) and 12 m (end of measurement). The time taken for the walk was measured manually using an electronic time watch for all participants and was additionally controlled with a light beam to trigger an additional watch (Wilhelm Köster Ingenieur für Zeitmessung, Ditzingen, Germany). The test was performed twice and mean speed calculated to avoid test-retest error. Prespecified secondary outcomes included the six minute walk test (in metres), Rivermead mobility index^18^ (range 0-15, with higher scores indicating better mobility), actigraphy for 24 hours (in steps daily, assessed with GT3x; Actigraph, Pensacola, FL), modified Rankin scale (mRS) score (a scale assessing disability after stroke; ranging from 0 for no symptoms to 6 for death), as well as measures of cognition, motor function, spasticity, mood, and sleep at follow-up compared with baseline. Additionally, in a per protocol dataset we analysed changes in maximal walking speed and Barthel index score three months after stroke compared with baseline. Prespecified biomarker and imaging outcome measures will be analysed separately within an accompanying study,^19^ as described in the statistical analysis plan. The supplementary appendix describes the assessments of secondary outcomes. Main prespecified subgroup analyses included dichotomisation of impairment scores (National Institutes of Health stroke scale (NIHSS)^20^ assessed on days 3-5 after stroke, a scale that measures neurological deficits, ranging from 0 to 42 points, with higher values indicating more severe strokes, and functional ambulation category), type of stroke, time of study inclusion, and age and sex of participants. We carried out additional post hoc exploratory analyses of differential treatment effects for continuous variables using splines.^21^

Primary safety outcomes included the serious adverse events of recurrent cardiovascular events, including stroke, admission to an acute care hospital, or death within three months after stroke. Trial physicians reported these outcomes to the coordinating trial centre within 24 hours. The adverse events of self reported pain, fatigue, dizziness, and number and nature of falls during the intervention period were recorded after each intervention session. We reported adverse events to the data safety monitoring board on a regular basis.

### Statistical analysis

To show the superiority of the aerobic physical fitness training over relaxation intervention, the study was powered on the primary outcome measures to detect a clinically meaningful difference of 0.13 m/s in maximal walking speed (common standard deviation of 0.25 m/s) or 10 points in Barthel index score (common standard deviation of 21 points).^14^ Assumptions were based on reported clinical differences from a previous study of another working group (n=155).^13^ Overall, 172 participants (86 in each group) were needed to provide 80% power to detect a statistically significant treatment effect for each of the primary endpoints (maximal walking speed and Barthel index score at three months compared with baseline; two sided significance level α=0.025 for each primary outcome, two sample t test). Accounting for a 20% dropout rate, we planned a total sample size of 215 participants. The predefined analyses have been performed as described in the statistical analyses plan of the trial (version 1.0, available online) and were conducted using SPSS, STATA, and R statistical software.^22^ ^23^ ^24^

All randomised participants were included in the full dataset for the intention-to-treat analysis. Group differences for each primary outcome were analysed using two separate analyses of covariance, with baseline measures as covariates and an additional random effect (random intercept model) to account for clustering of participants in centres. The primary outcomes at follow-up (maximal walking speed and Barthel index score three months after stroke) were the dependent variables in these analyses, and baseline scores and intervention group were independent variables. Additionally, we adjusted the analyses for age, sex, and functional impairment (as assessed by the functional ambulation category test). We imputed missing data because of attrition by using multivariate imputation by chained equations (mice) based on 10 imputed datasets and relevant information generated by the R package mice.^25^ The supplementary appendix provides detailed descriptions of data imputation and handling missing data. If we were unable to assess data on mobility measures at baseline because of severe impairment, reasonable single value imputation was carried out by using half the speed of the slowest participant in the group. Analyses of safety endpoints were done using Poisson regression models, which account for the time each participant is at risk and allows incidence rate and incidence rate ratios with confidence intervals to be calculated.

Prespecified subgroup analyses for the primary outcomes were exploratory for sex, age groups, type of stroke, impairment measured by NIHSS and functional ambulation category, and time from stroke to start of intervention. For each subgroup analysis, we tested the interaction between treatment allocation and subgroup to test whether any difference in treatment effect between subgroups was substantial. For each subgroup we also provided mean treatment effects and 95% confidence intervals.

Secondary outcome measures were analysed using a three level mixed model where the repeated measures were nested in participants and participants were nested in centres. We used baseline variables of outcomes as covariates. Models were additionally adjusted for age, sex, and baseline functional ambulation category. In all models we included interaction terms for time point and treatment group. Reported effect estimates were calculated using post-estimation procedures. All models (except for mRS and functional ambulation category) are based on 10 imputations with chained equations and groupwise imputation (see supplementary table S3). For the mRS and functional ambulation category, we used an ordinal logistic mixed regression model including respective covariates for adjustment and random intercept to account for repeated measures. The group variable for centre was entered as fixed covariate in the ordinal mixed models to avoid instability of models. Variables with non-normal distribution were log transformed before analyses. All secondary analyses were carried out in an exploratory framework. No adjustment for multiple testing was applied for secondary analyses.

### Patient and public involvement

Before and during the trial, a patient representative appointed by the Berlin Stroke Alliance participated in meetings of the trial’s executive steering committee. A member of the trial centre informed the Berlin Stroke Alliance (including participants and family members) about the progress of the trial on a regular basis. After the last trial visit, each participant received a summary of his or her personal outcome measures and blood test results. Findings of the trial will be shared with all participants by providing access to the full manuscript. The supplementary appendix provides a section for participants and carers who provided additional information.

## Results

**Fig 1:**
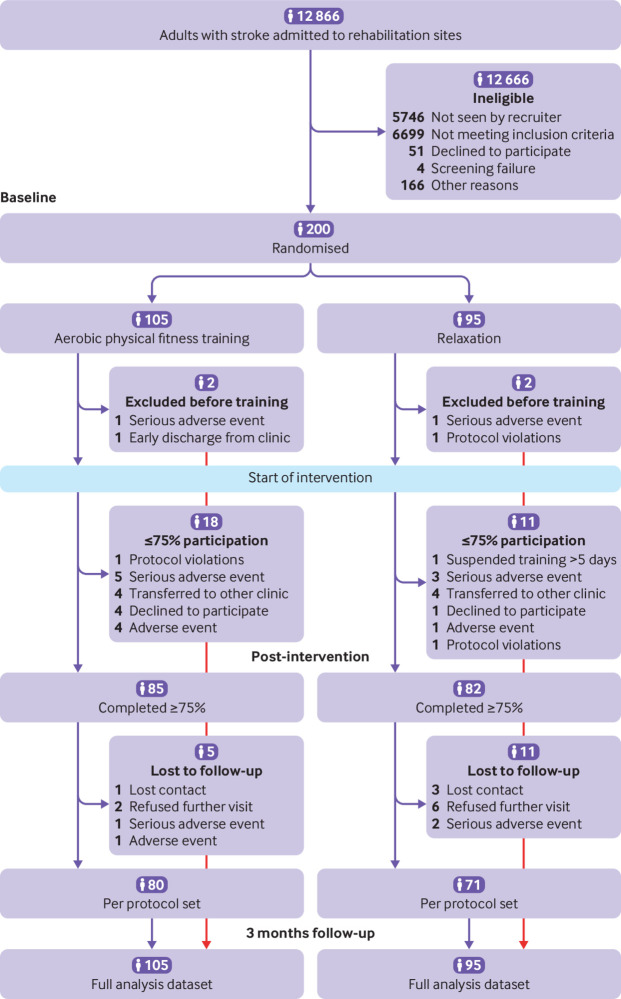
Flowchart of enrolment and randomisation. Multiple imputation was performed for intention-to-treat analyses of full analysis dataset

From 26 September 2013 to 30 April 2017, a total of 12 866 adults were screened of whom 200 were included in the trial and underwent randomisation (105 were assigned to aerobic physical fitness training and 95 to relaxation sessions (control group) in addition to standard care (fig 1). The main reasons for exclusion were a Barthel index score greater than 65, stroke onset more than 45 days before screening, and presence of comorbidities at the time of screening (mainly cardiac). The full analysis set entered the intention-to-treat analysis. The mean age of the study cohort was 69 (SD 12) years and 41% were women. The median NIHSS score was 8 (interquartile range 5-12) points at days 3-5 after stroke. Baseline personal and clinical characteristics were similar in both study groups, except for the six minute walk test and Rivermead mobility index, which were higher in the relaxation group. Table 1 lists the baseline characteristics. The amount of physiotherapy applied during inpatient and outpatient standard care between baseline and follow-up visit three months after stroke was similar between groups (see supplementary table S14).

**Table 1:** Baseline characteristics of participants stratified by aerobic physical fitness training or relaxation sessions (control group). Values are numbers (percentages) unless stated otherwise

Characteristic	Aerobic physical fitness training (n=105)	Relaxation sessions (n=95)	Total cohort (n=200)
Mean (SD) age (years)	69 (12)	70 (11)	69 (12)
Women	45 (43)	36 (38)	81 (41)
Median (interquartile range) time from stroke to intervention (days)*	30 (17-39)	27 (17-41)	28 (17-40)
Anterior circulation stroke	84 (80)	72 (76)	156 (78)
Hemiparesis on admission	98 (93)	89 (94)	188 (94)
Median (interquartile range) NIHSS score†	9 (5-12)	7 (5-11)	8 (5-12)
Ischaemic stroke	91 (87)	90 (95)	181 (91)
Treatment with alteplase‡	34 (37)	27 (30)	61 (34)
Cause of ischaemic stroke‡:			
Large artery atherosclerosis	17 (19)	19 (21)	36 (20)
Cardioembolism	18 (20)	18 (20)	36 (20)
Small vessel occlusion	16 (18)	15 (17)	31 (17)
Other causes	3 (3)	4 (4)	7 (4)
Undetermined causes	34 (37)	28 (31)	62 (34)
Competing causes	3 (3)	6 (7)	9 (5)
Previous stroke	27 (27)	27 (28)	54 (27)

NIHSS=National Institutes of Health stroke scale.

* Four participants were excluded at screening.

† Scores range from 0 to 42, with higher scores indicating greater stroke severity. Assessed on days 3-5 after stroke. Hospital chart was missing for one participant.

‡ Reported proportions of participants treated with alteplase and proportions of causes of stroke refer only to participants with ischaemic stroke.

**Fig 2:**
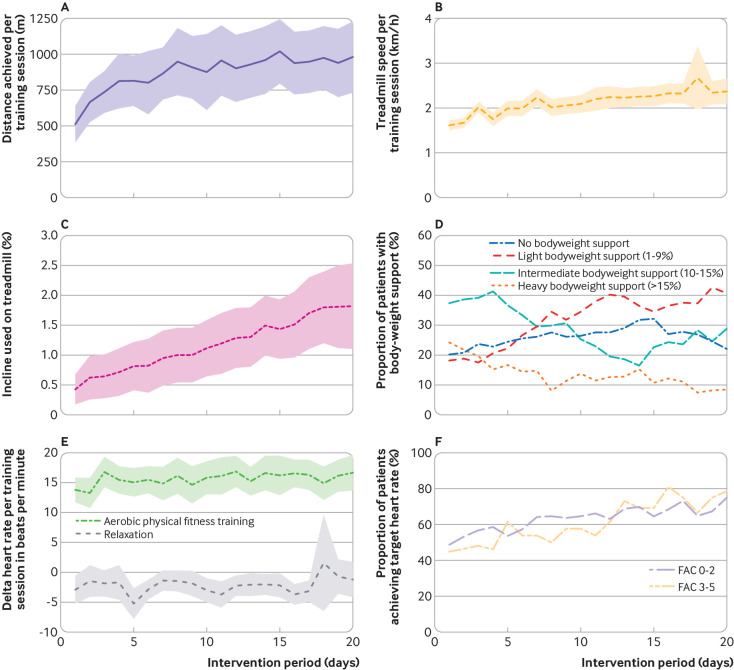
Progression of training modalities during intervention period. (A) Distance (m) achieved (only available for participants who used treadmill). (B) Walking speed (km/h) reached on treadmill. (C) Change in incline (%) on treadmill (only available for participants who used treadmill). (D) Proportion of participants with different levels of bodyweight support over time. (E) Mean change in heart rate by group measured before and after intervention sessions during intervention period. (F) Proportion of participants who achieved their target heart rate during training. Participants are grouped by baseline functional ambulation category (FAC) score of 0-2 and 3-5 (higher scores indicate less dependency)

The median time from stroke onset to start of intervention was 28 (interquartile range 17-40) days. Adherence to the study protocol was good to moderate (fig 2). The mean delta heart rate (pre-post intervention) was 15 (SD 9) beats per minute (bpm) in participants assigned to aerobic physical fitness training compared with −2 (SD 3) bpm in participants assigned to relaxation. Among participants randomised to aerobic physical fitness training, the median percentage of training sessions performed at target heart rate was 70% (interquartile range 23-100%) with improved adherence towards the end of the intervention period (fig 2). The amount of therapy applied during standard care was similar for severely impaired participants (functional ambulation category 0-1) compared with less severely impaired participants (>1), indicating similar treatment conditions (see supplementary table S7). Participants in the aerobic physical fitness training group received a mean of 16 (SD 6) sessions with a mean duration of the core intervention of 21 (SD 4) minutes, compared with a mean 17 (SD 5) sessions and mean duration of 24 (SD 3) minutes in the relaxation group. Eighteen participants in the aerobic physical fitness training group (17%) did not reach the required amount of 15 intervention sessions, compared with 11 participants (12%) in the relaxation group. Main reasons for termination of the intervention were early transfer to a non-participating hospital (n=4, each group) and a serious adverse event (n=5 in aerobic physical fitness training group; n=3 in relaxation group, see fig 1). At three months after stroke, 34 participants (17%) were lost to follow-up, and respective data were imputed for the intention-to-treat analysis using multiple imputation methods (see supplementary appendix for detailed description). The prespecified per protocol analysis comprised data for 151 participants (76% of the full analysis set), 80 participants in the aerobic physical fitness training group and 71 in the relaxation group.

**Fig 3:**
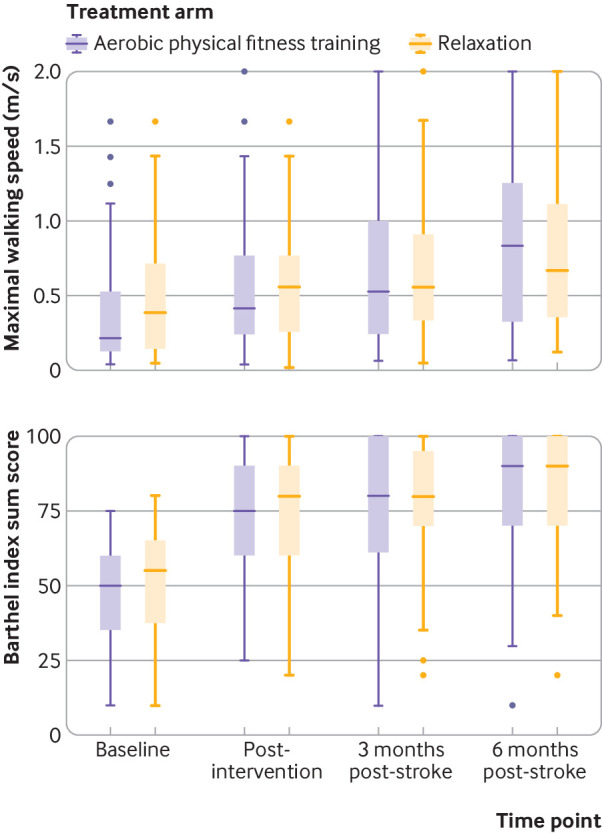
Boxplot showing medians (interquartile ranges) for maximal walking speed assessed by 10 m walk test (top panel), and Barthel index score (bottom panel) for each study visit and intervention group. Data are based on measurements without multiple imputation. Number of participants at each scheduled study visit was: baseline (n=105 in aerobic physical fitness training group, n=95 in relaxation group:), post-intervention (n=87, n=85), three months post-stroke (n=89, n=77), and six months post-stroke (n=80, n=65). Dots represent outliers

Table 2 lists the primary efficacy outcomes. The adjusted mean change of maximal walking speed from baseline to three months after stroke was 0.4 m/s (95% confidence interval 0.3 to 0.4 m/s) in the aerobic physical fitness training group and 0.3 m/s (0.2 to 0.4 m/s) in the relaxation group, resulting in an adjusted treatment effect of 0.1 m/s (0.0 to 0.2 m/s; P=0.23, primary efficacy outcome). The adjusted mean change in Barthel index score three months after stroke was 30 points (95% confidence interval 24 to 36) in the aerobic physical fitness training group compared with 30 points (23 to 36) in the relaxation group, resulting in an adjusted treatment effect of 0 (95% confidence interval −5 to 5; P=0.99, primary efficacy outcome). Figure 3 shows the change in maximal walking speed and Barthel index score during the trial.

**Table 2:** Results for primary efficacy outcome of change in maximal walking speed and Barthel index score from baseline to three months after stroke by aerobic physical fitness training or relaxation sessions (control group)

Primary outcomes	Aerobic physical fitness training (n=105)	Relaxation sessions (n=95)	Adjusted treatment effect*	P value
Mean (95% CI) maximal walking speed (m/s)	0.4 (0.3 to 0.4)	0.3 (0.2 to 0.4)	0.1 (0.0 to 0.2)	0.23
Mean (95% CI) Barthel index score	30 (24 to 36)	30 (23 to 36)	0 (−5 to 5)	0.99

Analyses based on multiple imputation.

* Treatment effects were analysed using analysis of covariance mixed models with three month outcome as dependent variable adjusted for baseline and additionally adjusted for sex, study centre, and functional ambulation category.

**Fig 4:**
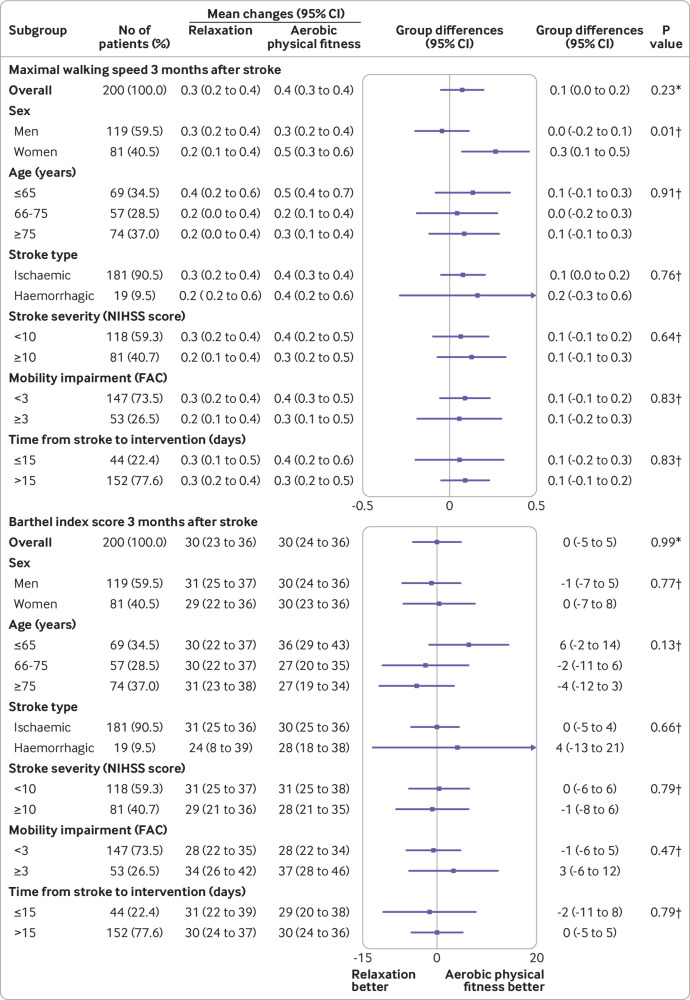
Prespecified subgroup analyses. Forest plots display maximal walking speed and Barthel index scores. Results are based on multiple imputation. No data were available for time from stroke to intervention for four participants who were excluded at screening. National Institutes of Health Stroke scale (NIHSS) score was missing for one participant owing to missing hospital chart. FAC=functional ambulation category. *P value for primary outcome measure. †P values for age×group interaction

**Fig 5:**
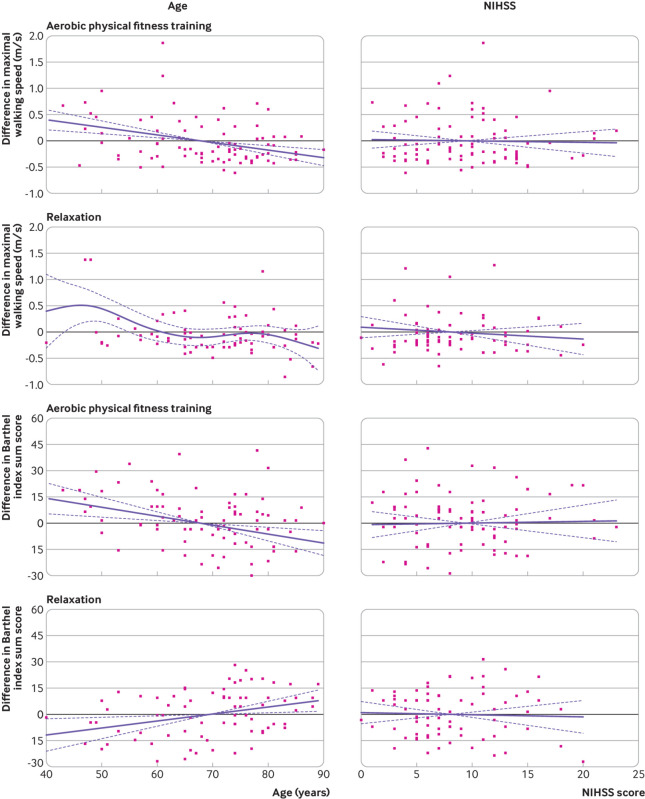
Subgroup analyses of continuous variables using splines. Differences in maximal walking speed and Barthel index scores (follow-up three months after stroke-baseline) as a function of age and National Institutes of Health stroke scale (NIHSS) score at days 3-5 after stroke

In the per protocol analysis, the mean change in maximal walking speed was 0.4 m/s (95% confidence interval 0.3 to 0.5 m/s) in the aerobic physical fitness training group and 0.3 m/s (0.2 to 0.4 m/s) in the relaxation group, resulting in a treatment effect of 0.1 m/s (95% confidence interval −0.1 to 0.2 m/s). The mean increase in Barthel index score after three months was similar in both groups: aerobic physical fitness training 32 (95% confidence interval 28 to 37) versus relaxation 31 (27 to 35); treatment effect 1 (95% confidence interval −4 to 6). Figure 4 shows the results for the prespecified subgroup analyses. Exploratory subgroup analysis for maximal walking speed suggested a greater treatment effect in women than in men: 0.3 (95% confidence interval 0.1 to 0.5) *v* 0.0 ( −0.2 to 0.1); P=0.01 for interaction. Figure 5 and supplementary figure S2 show the results for subgroup analyses of continuous variables using splines.

All secondary analyses were done in an exploratory framework. Overall, no substantial differences in maximal walking speed or Barthel index score between groups were observed at the end of intervention or at follow-up six months after stroke (table 3).

**Table 3:** Results for secondary outcome measures by aerobic physical fitness training or relaxation sessions (control group)

Secondary outcomes by groups	Baseline: aerobic physical fitness training (n=105), relaxation sessions (n=95)		Post-intervention: aerobic physical fitness training (n=87), relaxation sessions (n=85)		Follow-up			Treatment effect (95%CI)*		
					3 months: aerobic physical fitness training (n=89), relaxation sessions (n=77)	6 months: aerobic physical fitness training (n=79), relaxation sessions (n=65)		Post-intervention	3 months follow-up	6 months follow-up
**Median (interquartile range); mean (SD) maximal walking speed (m/s)†**										
Aerobic training	0.2 (0.1-0.5); 0.4 (0.4)		0.4 (0.2-0.8); 0.6 (0.5)		0.6 (0.3-1.1); 0.8 (0.6)	0.8 (0.3-1.3); 0.9 (0.8)		0.03 (−0.10 to 0.16)	Primary outcome	0.09 (−0.04 to 0.22)
Relaxation	0.3 (0.1-0.7); 0.5 (0.4)		0.6 (0.3-0.8); 0.6 (0.6)		0.6 (0.4-0.9); 0.8 (0.7)	0.8 (0.4-1.3); 0.9 (0.9)				
**Median (interquartile range); mean (SD) Barthel index score**										
Aerobic training	50 (35-60); 47 (16)		75 (60-90) 73 (21)		80 (61-100); 77 (22)	90 (70-100); 82 (20)		0 (−4 to 5)	Primary outcome	−1 (−6 to 3)
Relaxation	55 (35-65); 49 (17)		80 (60-90); 75 (22)		80 (70-95); 79 (19)	90 (70-100); 84 (18)				
**Median (interquartile range); mean (SD) 6 minute walk distance (m)‡**										
Aerobic training	75 (32-160); 107 (110)		145 (85-245); 175 (126) (n=81)		165 (90-300); 201 (153) (n=85)	220 (110-350); 239 (152) (n=77)		19 (−8 to 46)	27 (0 to 54)	26 (−1 to 53)
Relaxation	120 (39-205); 139 (113)		179 (91-244); 185 (115) (n=78)		180 (110-263); 203 (128) (n=71)	208; (114-323) 233 (149) (n=64)				
**Mean (SD) Rivermead mobility index score**										
Aerobic training	5 (3)		8 (4)		9 (4)	11 (4)		0.2 (−0.6 to 1.0)	0.3 (−0.5 to 1.1)	0.0 (−0.8 to 0.8)
Relaxation	6 (3)		9 (4)		9 (4)	11 (4) (n=65)				
**Median (interquartile range) modified Ranking scale score**										
Aerobic training	4 (4-4)		4 (3-4)		3 (2-4) (n=90)	3 (2-4)		2.0 (0.6 to 6.9)§	0.8 (0.2 to 2.5)§	1.1 (0.3 to 3.6)§
Relaxation	4 (3-4)		3 (3-4)		3 (3-4) (n=78)	3 (2-4)				
**Median (interquartile range) actigraphy (steps/day)¶**										
Aerobic training	3263 (1815-5515); (n=97)		4758 (2910-7056); (n=70)		4215 (2042-6399); (n=77)	4284 (2193-7308); (n=63)		−555 (−1486 to 375)	−539 (−1467 to 394)	−566 (−1497 to 365)
Relaxation	3503 (1949-6328); (n=88)		5183 (2945-7876); (n=75)		5160 (3194-7980); (n=69)	6105 (3404-7904); (n=53)				
Median (interquartile range) step length (m)**										
Aerobic training	0.31 (0.23-0.44); (n=99)		0.39 (0.30-0.51); (n=85)		0.43 (0.29-0.56); (n=88)	0.50 (0.35-0.63); (n=78)		0.04 (−0.01 to 0.07)	0.03 (−0.02 to 0.07)	0.03 (−0.01 to 0.07)
Relaxation	0.39 (0.29-0.48); (n=93)		0.40 (0.30-0.50); (n=83)		0.42 (0.34-0.56); (n=76)	0.47 (0.33-0.64); (n=64)				
**No (%) used walking aid**										
Aerobic training	69 (75) (n=92)		57 (71) (n=80)		55 (63) (n=88)	48 (60) (n=79)		0.46§ (0.10 to 2.19)	0.29§ (0.06 to 1.41)	0.30§ (0.06 to 1.57)
Relaxation	60 (78) (n=77)		59 (76) (n=78)		52 (72) (n=72)	42 (68) (n=62)				
**Median (interquartile range) step cadence (steps/min)††**										
Aerobic training	53 (29-91); (n=99)		68 (46-102); (n=84)		91 (54-116); (n=88)	100 (59-128); (n=78)		2 (−7 to 12)	6 (−4 to 15)	3 (−7 to 12)
Relaxation	70 (36-92); (n=93)		80 (57-99); (n=83)		90 (65-113); (n=76)	98 (68-126); (n=64)				
**Median (interquartile range) box and block test, impaired hand/non-impaired hand‡‡**										
Aerobic training	0 (0-31) / 46 (34-54) (n=100)		12 (0-38) / 48 (40-58) /		19 (0-39) / 53 (40-62)	27 (0-44) / 54 (42-62)		1 (−3 to 6)	1 (−3 to 5)	−1 (−6 to 3)
Relaxation	2 (0-23) / 45 (35-52) (n=89)		12 (0-34) / 46 (39-56) /		23 (0-35) / 45 (38-56)	28 (3-43) / 50 (39-60)				
**Mean (SD) Rivermead mobility index score: subtest arm**										
Aerobic training	5 (5)		6 (6)		6 (6)	8 (6)		−0.7 (−1.6 to 0.2)	−0.9 (−1.8 to 0.0)	−1.1 (−2.0 to 0.0)
Relaxation	5 (5)		7 (5)		8 (5)	9 (5)				
**Mean (SD) medical research council scale, sum score over six items§§**										
Aerobic training	19 (8) (n=104)		22 (7) (n=86)		22 (6)	23 (7)		0.3 (−0.8 to 1.4)	−0.3 (−1.4 to 0.8)	−0.2 (−1.3 to 0.9)
Relaxation	20 (8)		22 (7) (n=85)		23 (7)	24 (5)				
**Mean (SD) resistance to passive movement scale sum score**										
Aerobic training	4 (5)		6 (6)		8 (9) (n=88)	11 (14)		0.9 (−1.0 to 2.9)	1.6 (−0.3 to 3.6)	2.6 (0.6 to 4.5)
Relaxation	4 (5)		5 (6)		6 (7) (n=77)	9 (9)				
**Median (interquartile range ) functional ambulation category score¶¶**										
Aerobic training	2 (1-2)		3 (2-3) (n=56)		3 (2-4) (n=52)	4 (2-5) (n=47)		0.3 (0.1 to 1.6)§	0.9 (0.2 to 5.0)§	1.1 (0.2 to 6.2)§
Relaxation	2 (1-3)		3 (2-4) (n=57)		3 (2-4) (n=44)	3 (3-4) (n=33)				
Median (interquartile range) gait energy cost (ml/kg^-1^/m^-1^)***										
Aerobic training	0.8 (0.4-1.2) (n=54)		0.4 (0.3-0.6) (n=51)		0.4 (0.3-0.6) (n=49)	0.3 (0.2-0.5) (n=35)		−0.1 (−0.2 to 0.0)	−0.1 (−0.2 to 0.0)	−0.1 (−0.2 to 0.0)
Relaxation	0.4 (0.3-0.7) (n=46)		0.4 (0.3-0.5) (n=41)		0.4 (0.3-0.6) (n=41)	0.4 (0.3-0.5) (n=32)				
**Mean (SD) quality of life (EQ-5D-5L) index score†††**										
Aerobic training	0.5 (0.3) (n=104)		0.7 (0.3) (n=87)		0.7 (0.3) (n=87)	0.7 (0.3)		0.04 (−0.04 to 0.11)	0.03 (−0.05 to 0.11)	0.0 (−0.08 to 0.08)
Relaxation	0.5 (0.3) (n=93)		0.7 (0.3) (n=82)		0.6 (0.3) (n=77)	0.7 (0.3)				
**Mean (SD) depression (CES-D) sum score‡‡‡**										
Aerobic training	10 (7) (n=85)		9 (6) (n=72)		10 (7) (n=71)	8 (7)		−1 (−3 to 1)	0 (−2 to 2)	0 (−2 to 1)
Relaxation	10 (5) (n=72)		10 (5) (n=67)		10 (6) (n=64)	9 (5)				
**Mean (SD) sleep (PSQI) sum score§§§**										
Aerobic training	4 (3) (n=96)		5 (4) (n=83)		6 (3) (n=84)	5 (4) (n=77)		−1 (−2 to 0)	−1 (−1 to 0)	−1 (−1 to 0)
Relaxation	5 (3) (n=88)		6 (4) (n=78)		6 (4) (n=74)	5 (3) (n=60)				
**Median (interquartile range) cognition (MOCA) sum score¶¶¶**										
Aerobic training	24 (21-27) (n=104)		26 (22-28) (n=86)		25 (22-28) (n=89)	26 (24-29)		0 (−1 to 1)	−1 (−1 to 1)	0 (−1 to 1)
Relaxation	24 (17-26) (n=94)		25 (19-27) (n=84)		25 (21-28) (n=75)	26 (21-28) (n=64)				
Median (interquartile range) cognitive processing speed (TMT A) (sec)****										
Aerobic training	70 (52-122) (n=104)		56 (42-120) (n=85)		51 (37-84)	52 (33-69)		5 (−10 to 20)	7 (−7 to 22)	1 (−14 to 16)
Relaxation	85 (60-197) (n=94)		64 (47-123) (n=84)		67 (43-102)	60 (42-82)				
**Median (interquartile range) executive functioning (TMT B) (sec)††††**										
Aerobic training	222 (127-301) (n=104)		97 (93-301) (n=85)		139 (88-301)	125 (78-216)		−5 (−21 to 11)	−1 (−17 to 16)	−6 (−22 to 10)
Relaxation	301 (157-301) (n=93)		218 (134-301) (n=84)		166 (110-301)	150 (92-301)				
**Mean (SD) word fluency (RWT) sum score‡‡‡‡**										
Aerobic training	38 (19) (n=104)		-		43 (19) (n=86)	-		-	1 (−2 to 3)	-
Relaxation	34 (18) (n=92)		-		40 (18) (n=73)	-				

Analyses are based on mixed models analysis of covariance (adjusted for baseline value, age, sex, functional ambulation category, and centre heterogeneity). Estimates are based on three level mixed models and multiple imputation (n=600 measures, 200 participants, six study centres) positive values favour aerobic physical fitness training. Missing values were imputed by multiple imputation, except for modified Ranking scale score, use of walking aids, and functional ambulation category (imputation process was done separately for treatment groups). If the number of valid data points differs for specific variables, the number of available data points is listed in brackets. Data missing due to missing at random are imputed (see supplementary appendix).

* Secondary outcomes are exploratory and not meant for hypothesis testing. P values are therefore not reported.

† 21 participants were unable to walk 10 m to assess maximal walking speed—values were therefore imputed using single value imputation by taking half of lowest value of total cohort.

‡ 17 participants were unable to walk for entire time of six minutes—for those participants distance walked up to stopping is used. 28 participants were in poor physical condition and could not do the test—values were therefore imputed, using single value imputation by taking half of lowest value of total cohort.

§ Difference between groups at three months after stroke presented as odds ratios (95% confidence intervals, odds ratio >1 favours aerobic training intervention. Calculations not adjusted for centre heterogeneity. For walking aids odds ratio >1 means dependence on walking aids in aerobic training group. Table 2 shows results for primary efficacy outcomes.

¶ 15 baseline actigraphy values were missing due to logistic reasons. Data are assumed to be missing completely at random.

** Missing values in step length are due to implausible number of steps within 10 m gait assessment, or 10 m walk test not completed.

†† Missing values in step cadence are due to implausible number of steps within 10 m gait assessment, or 10 m walk test not completed.

‡‡ 11 participants showed no initial motor impairment and are excluded from analysis.

§§ One baseline value on medical research council scale is missing at random in aerobic group.

¶¶ Functional ambulation category was initially assessed at baseline only and not at follow-up; therefore some follow-up values are missing.

*** Spirometry data are missing mostly due to technical issues. Baseline values are available for 100 participants (n=54 aerobic training, n=46 relaxation).

††† Six baseline values for EuroQol quality of life questionnaire (EQ-5D-5L) index score were missing for various reasons (aphasia, fatigue, understanding difficulties).

‡‡‡ 19 baseline values for Center for Epidemiologic Studies depression scale (CES-D) sum score were missing owing to aphasia, fatigue, understanding difficulties, not able to respond to question. 24 data points had to be excluded because participants fulfilled criteria for unreliable data (lie, criteria ≤28).

§§§ 16 baseline values for Pittsburgh sleep quality index (PSQI) sum score were missing owing to aphasia, fatigue, and understanding difficulties.

¶¶¶ Two baseline values for Montreal cognitive assessment (MOCA) sum score were missing owing to fatigue.

**** Two baseline values for trail making test (TMT) part A were missing owing to poor vision and fatigue.

†††† Three baseline values for trail making test (TMT) part B were missing owing poor vision, fatigue, and failure of time recording by assessor.

‡‡‡‡ The Regensburger Wortflüssigkeitstest (RWT) was only assessed at baseline and at three months’ follow-up. Four baseline values for RWT were missing owing to severe aphasia.

The six minute walk test showed a mean adjusted difference of 27 m (95% confidence interval 0 to 54 m) in favour of aerobic physical fitness training compared with relaxation at three months after stroke (adjusted for baseline, age, sex, and functional ambulation category). Other exploratory secondary analyses did not show substantial differences between study groups three months after stroke. At follow-up six months after stroke, the relaxation group showed greater improvements in the resistance to passive movement scale sum score (treatment effect 2.6 (95% confidence interval 0.6 to 4.5)) and the Rivermead mobility index subtest arm (treatment effect −1.1 (−2.0 to 0.0)) compared with aerobic physical fitness training (table 3).

The aerobic physical fitness training group had a higher rate of serious adverse events (n=22) than the relaxation group (n=9) at three months after stroke (incidence rate ratio 1.81, 95% confidence interval 0.97 to 3.36). Fourteen participants in the aerobic physical fitness training group were admitted to acute hospitals compared with five in the relaxation group (2.53, 0.91 to 7.04). Recurrent strokes occurred in eight participants in the aerobic physical fitness training group and in three participants in the relaxation group (2.41, 0.64 to 9.10). No serious adverse events occurred during intervention sessions. Self reported falls during the intervention period occurred more often in the aerobic physical fitness training group (2.34, 1.26 to 4.34) and self reported dizziness occurred more often in the relaxation group (0.33, 0.12 to 0.90, table 4).

**Table 4:** Safety outcomes by aerobic physical fitness training or relaxation session (control group)

Events	Aerobic physical fitness training (n=105)	Relaxation sessions (n=95)	Total cohort (n=200)	Incidence rate ratio (95% CI)*
**Serious adverse events† (from baseline to three months after stroke)**				
Median (interquartile range) follow-up (days)	68 (56-78)	69 (54-77)	66 (56-78)	
Total No	22	9	31	
Incidence rate/100 person months (95% CI)	13.19 (9.22 to 18.86)	7.28 (4.39 to 12.08)	10.38 (7.75 to 13.90)	1.81 (0.97 to 3.36)
Cardiovascular event	0	0	0	-
No with recurrent stroke	8	3	11	-
Incidence rate/100 person months (95% CI)	3.52 (1.76 to 7.03)	1.46 (0.47 to 4.52)	2.54 (1.41 to 4.58)	2.41 (0.64 to 9.10)
No of hospital admissions	14	5	19	-
Incidence rate/100 person months (95% CI)	6.15 (3.64 to 10.39)	2.43 (1.01 to 5.83)	4.39 (2.80 to 6.87)	2.53 (0.91 to 7.04)
No of deaths	0	1	1	-
Incidence rate/100 person months (95% CI)	-	0.49 (0.07 to 3.45)	0.23 (0.03 to 1.64)	0.30 (0.01 to 7.42)‡
**Self reported adverse events§ (during intervention period)**				
Median (interquartile range) follow-up time (days)	33 (29-36)	22 (28-36)	33 (29-36)	
No of falls	36	14	50	-
Incidence rate/100 person months (95% CI)	32.40 (23.37 to 44.92)	13.84 (8.19 to 23.36)	11.8 (8.9 to 15.5)	2.34 (1.26 to 4.34)
No of fractures	0	0	0	-
No with pain	68	44	112	-
Incidence rate/100 person months (95% CI)	61.20 (48.25 to 77.62)	43.49 (32.36 to 58.44)	26.1 (21.7 to 31.4)	1.41 (0.96 to 2.06)
No with fatigue	29	21	50	-
Incidence rate/100 person months (95% CI)	26.10 (18.14 to 37.56)	20.76 (13.53 to 31.83)	23.55 (17.85 to 31.07)	1.26 (0.72 to 2.20)
No with dizziness	5	14	19	-
Incidence rate/100 person months (95% CI)	4.50 (1.87 to 10.81)	13.84 (8.19 to 23.36)	8.95 (5.71 to 14.03)	0.33 (0.12 to 0.90)
Other	8	4	12	-
Incidence rate/100 person months (95% CI)	7.20 (3.60 to 14.40)	3.95 (1.48 to 10.53)	5.65 (3.21 to 9.95)	1.82 (0.55 to 6.05)

* Calculated using Poisson regression.

† Serious adverse event can belong to more than one category (eg, recurrent stroke and hospital admission). Thus total number of participants with a serious adverse event does not equal sum of individual categories of serious adverse events.

‡ Calculated using penalised maximum likelihood logistic regression (firthlogit command in stata).

§ Adverse events can occur multiple times in a participant. Time interval ranges from baseline to follow-up at three months after stroke or until last observation, if participant dropped out.

## Discussion

In adults with moderate to severe subacute stroke receiving standard inpatient rehabilitation therapy, a four week intervention of additional aerobic physical fitness training was not superior to a control intervention based on relaxation in improving activities of daily living and maximal walking speed three months after stroke. Analysis of safety showed an increased risk of falls during the treatment period and suggested a higher number of acute hospital admissions and recurrent strokes in participants randomised to aerobic physical fitness training. For clinical practice, the results of this pragmatic trial^26^ do not support the use of aerobic physical fitness training in moderately or severely affected adults in the subacute phase of stroke.

### Comparison with other studies

The treatment effects in mobility outcomes observed in the current study are in line with results reported in the latest Cochrane Collaboration meta-analysis of physical fitness trials after stroke^6^ and randomised trials of circuit class training interventions.^27^ ^28^ In our trial, however, neither the intention-to-treat analysis nor the per protocol analysis showed a substantial difference in maximal walking speed between study groups. This finding contrasts with that of previously published trials reporting beneficial effects of fitness training on maximal walking speed.^29^ ^30^ ^31^ Interventions in these previous trials were compared with usual care only, however, and the reported improvements were in smaller cohorts comprising participants with late subacute or chronic stroke and less severe disability. As recently endorsed by the Stroke Recovery and Rehabilitation Roundtable taskforce, trials should focus on the early subacute phase of stroke (days 7-90 after stroke)^10^; in the present trial more than 99% of participants were recruited in this subacute phase. This phase is thought to be a critical time for neuroplasticity^32^ and therefore might serve as an important period for rehabilitation to harness endogenous neural repair. Despite early enrolment of participants to our trial, we were not able to show enhanced treatment effects. Interestingly, a recent meta-analysis of randomised controlled trials on bodyweight supported treadmill training after stroke concluded that participants who walked independently (functional ambulation category score >2) improved in walking speed and walking endurance, whereas those who were not able to walk independently (score 0-2) did not improve with treadmill training.^33^ This fact could explain why our trial failed to show a statistically significant improvement in primary outcome measures, given that about 75% of the study cohort had functional ambulation category scores between 0 and 2 at baseline. However, no interaction between functional ambulation category score and treatment effect was observed in the respective subgroup analysis (fig 4 and supplementary figure S2). No treatment effect was observed with activities of daily living three months after stroke, which contrasts with the findings of smaller, previous trials including participants in the early and late subacute stage of stroke.^12^ ^13^ Until now, evidence that physical fitness reduces disability after stroke has been inconclusive for activities of daily living, and positive effects on disability after stroke could primarily derive from improvements in mobility.^6^ Aerobic physical fitness training compared with relaxation had no beneficial effect on scores on the Barthel index, functional ambulation category, or modified Rankin scale (see tables 2 and 3). Current outcome measures of activities of daily living differ in sensitivity, and the Barthel index might not be sensitive enough to capture small but clinically meaningful differences in activities of daily living related to aerobic training after stroke. Exploratory analyses of secondary outcome measures suggested small beneficial effects in fitness measures for participants randomised to aerobic physical fitness training at three months after stroke, as reflected in the six minute walk test (see table 3). This was a secondary exploratory finding, however, and cannot be used for inferences on treatment effects. Thus, adequately powered trials need to confirm whether bodyweight supported, treadmill based, aerobic physical fitness training improves measures of endurance at a clinically important level in this stroke population.

No safety concerns were reported for aerobic physical fitness training in the early subacute phase in two smaller previous studies comprising survivors of mild to moderate stroke.^34^ ^35^ However, in the large randomised controlled LEAPS trial, the proportion of participants with multiple falls was higher in the early locomotor training group (starting two months after stroke) compared with the late locomotor training group (starting six months after stroke).^11^ Similarly, in the current trial, falls occurred more often in the aerobic physical fitness training group than in the relaxation group during the intervention period. In contrast, participants in the relaxation group reported dizziness more often than participants in the aerobic physical fitness training group (see table 4). People with stroke are at increased risk for repeat falls, and fall prevention programmes are needed.^36^ Hence, and in opposition to current guideline recommendations,^7^ treadmill based aerobic physical fitness training should be administered with caution early after moderate to severe stroke. A Very Early Rehabilitation Trial after stroke (AVERT) mobilised participants within 24 hours after stroke and found a worse outcome after three months, but no differences were observed in walking related outcome measures.^37^ Although AVERT, LEAPS, and the present trial differ in terms of study population, timing after stroke, and type of intervention, these studies did not replicate the previously reported beneficial effects of specific rehabilitation interventions in small cohorts, while at the same time detecting a larger number of relevant adverse events and serious adverse events.

### Strengths and limitations of this study

This is a randomised controlled, multicentre stroke rehabilitation trial assessing the efficacy and safety of an aerobic, bodyweight supported fitness intervention compared with relaxation in adults with subacute stroke. Compared with previous post-stroke fitness trials,^6^ our trial recruited a substantially larger sample size comprising adults with moderate to severe stroke and assessed a broad spectrum of patient centred outcome measures. The study does, however, have limitations. Firstly, recruitment took place between days 5 and 45 after stroke, therefore variations in early neurological recovery could have increased differences in outcome measures and might have affected current conclusions. However, the impact of the additionally induced variations is limited because almost all participants were recruited within the time window of early subacute stroke,^10^ and subgroup analyses (see fig 4) could not detect differences in treatment effects for participants receiving the intervention early after stroke (<2 weeks) compared with late after stroke (>2 weeks). Secondly, despite random allocation, the aerobic physical fitness training group was more severely affected at baseline, but we adjusted for baseline values in statistical analyses. Thirdly, findings are only applicable to moderately to severely affected adults with subacute stroke and are not generalisable to the stroke population, especially not to people with chronic stroke. Fourthly, less than 4% of the screened adults with stroke were included in the trial, and the severity of impairment might have resulted in a substantial proportion of participants terminating the intervention prematurely; a limitation often observed in early stroke rehabilitation trials.^9^ ^11^ ^38^ Fifthly, the intervention period of four weeks could have been too short to show additional benefits compared with a relaxation control intervention; and the lack of a control arm for usual care only limits the understanding of the treatment effects of relaxation therapy.

### Clinical implications and future research

Our trial provides evidence that an aerobic physical fitness training intervention with bodyweight support cannot be generally endorsed in adults after subacute stroke and should be administered with caution when applied early after moderate or severe stroke. Carers should closely monitor people with stroke for recurrent cardiovascular events and provide additional support after training to prevent falls. Despite these findings, aerobic physical fitness could still be an invaluable part of stroke rehabilitation—for example, in people with chronic stroke or mildly affected with subacute stroke.

Based on our results, trials should investigate if longer intervention periods are necessary to capture major changes in activities of daily living, assess the treatment effects of relaxation programmes compared with usual care, and recruit participants at fixed time points after stroke to reduce variance in outcome measures.^10^ ^39^ Future trials might also evaluate whether the observed sex specific difference in change of maximal walking speed or the effects on endurance measures can be replicated.

### Conclusion and policy implications

A four week intervention of a bodyweight supported, treadmill based, aerobic physical fitness training in adults in the subacute phase of moderate to severe stroke is not superior to relaxation sessions with regard to maximal walking speed and activities of daily living. The risk of falls was higher in participants randomised to aerobic physical fitness training. Compared with current guideline recommendations,^7^ these results do not appear to support the use of aerobic bodyweight supported fitness training in this stroke population.

**What is already known on this topic**

Current guidelines endorse cardiorespiratory training within post-stroke rehabilitation programmes
Large randomised controlled trials of this recommendation are scarce, resulting in inconclusive data on efficacy for disability (activities of daily living) and safety of physical fitness training after stroke

**What this study adds**

Results suggest that in adults with moderate to severe subacute stroke, in addition to standard rehabilitation care, aerobic, bodyweight supported, treadmill based fitness training did not improve maximal walking speed or activities of daily living compared with relaxation
The rate of serious adverse events was higher in the aerobic physical fitness training group than relaxation group
Compared with current guideline recommendations, these results do not appear to support the use of aerobic, bodyweight supported, treadmill based fitness training in this stroke population

## Web extra

Extra material supplied by authors

**Supplementary material:** inclusion and exclusion criteria

## Notes

Contributors: AHN, TR, MEb, and AF contributed equally to the study. AF, MEb, AHN, TR, AM, and SH conceived or designed the study. All authors were responsible for acquisition of data, data analysis, or interpretation of data. AHN drafted the manuscript. AF, AHN, MEb, and TR had full access to all the data in the study and take responsibility for the integrity of the data and the accuracy of the data analysis. AHN, TR, MEb, and AF contributed equally to the final manuscript. UG and TR performed statistical analyses. AF, MEb, ME obtained funding. AM and ME provided administrative, technical, or material support. AF, AHN, MEb, and TR supervised the study. All authors critically revised the manuscript for important intellectual content and gave final approval of the version to be published. AF is the guarantor of the paper and takes responsibility for the integrity of the work from inception to published article. The corresponding author attests that all listed authors meet authorship criteria and that no others meeting the criteria have been omitted. SH died before publication of the study.

Ethical approval: This study was approved by the institutional review board of Charité–Universitätsmedizin Berlin (EA1/138/13). The trial was conducted in line with the CONSORT extension for non-drug treatments, and study procedures were carried out in accordance with the Declaration of Helsinki.

Data sharing: The statistical analysis plan is available at https://doi.org/10.6084/m9.figshare.5375026.v1. The raw trial data are provided by the authors on a secure online repository (see supplementary appendix for link). Data include anonymised individual patient variables for results reported here, a read-me file with data dictionary and analyses scripts used in this study. Data will be available three months after publication of the article. Further information can be shared on individual request addressed to the corresponding author.

The lead authors (AHN, TR, MEb, and AF) affirm that the manuscript is an honest, accurate, and transparent account of the study being reported; that no important aspects of the study have been omitted; and that any discrepancies from the study as planned (and, if relevant, registered) have been explained.

## References

[1] Catanese L Tarsia J Fisher M Acute Ischemic Stroke Therapy Overview. Circ Res 2017;120:541-58. 10.1161/CIRCRESAHA.116.309278 28154103

[2] Feigin VL Norrving B Mensah GA Global Burden of Stroke. Circ Res 2017;120:439-48. 10.1161/CIRCRESAHA.116.308413 28154096

[3] Wolfe CDA Crichton SL Heuschmann PU Estimates of outcomes up to ten years after stroke: analysis from the prospective South London Stroke Register. PLoS Med 2011;8:e1001033. 10.1371/journal.pmed.1001033 21610863 PMC3096613

[4] Crichton SL Bray BD McKevitt C Rudd AG Wolfe CD Patient outcomes up to 15 years after stroke: survival, disability, quality of life, cognition and mental health. J Neurol Neurosurg Psychiatry 2016;87:1091-8. 10.1136/jnnp-2016-313361 27451353

[5] Langhorne P Bernhardt J Kwakkel G Stroke rehabilitation. Lancet 2011;377:1693-702. 10.1016/S0140-6736(11)60325-5 21571152

[6] Saunders DH, Sanderson M, Hayes S, et al. Physical fitness training for stroke patients. *Cochrane Database of Systematic Reviews* 2016;(3):CD003316. 10.1002/14651858.CD003316.pub6 PMC6464717 27010219

[7] Billinger SA Arena R Bernhardt J American Heart Association Stroke Council Council on Cardiovascular and Stroke Nursing Council on Lifestyle and Cardiometabolic Health Council on Epidemiology and Prevention Council on Clinical Cardiology Physical activity and exercise recommendations for stroke survivors: a statement for healthcare professionals from the American Heart Association/American Stroke Association. Stroke 2014;45:2532-53. 10.1161/STR.0000000000000022 24846875

[8] Langhorne P Coupar F Pollock A Motor recovery after stroke: a systematic review. Lancet Neurol 2009;8:741-54. 10.1016/S1474-4422(09)70150-4 19608100

[9] Bernhardt J Godecke E Johnson L Langhorne P Early rehabilitation after stroke. Curr Opin Neurol 2017;30:48-54. 10.1097/WCO.0000000000000404 27845945

[10] Bernhardt J Hayward KS Kwakkel G Agreed definitions and a shared vision for new standards in stroke recovery research: The Stroke Recovery and Rehabilitation Roundtable taskforce. Int J Stroke 2017;12:444-50. 10.1177/1747493017711816 28697708

[11] Duncan PW Sullivan KJ Behrman AL LEAPS Investigative Team Body-weight-supported treadmill rehabilitation after stroke. N Engl J Med 2011;364:2026-36. 10.1056/NEJMoa1010790 21612471 PMC3175688

[12] Wang Z Wang L Fan H Lu X Wang T Effect of low-intensity ergometer aerobic training on glucose tolerance in severely impaired nondiabetic stroke patients. J Stroke Cerebrovasc Dis 2014;23:e187-93. 10.1016/j.jstrokecerebrovasdis.2013.09.021 24231135

[13] Pohl M Werner C Holzgraefe M Repetitive locomotor training and physiotherapy improve walking and basic activities of daily living after stroke: a single-blind, randomized multicentre trial (DEutsche GAngtrainerStudie, DEGAS). Clin Rehabil 2007;21:17-27. 10.1177/0269215506071281 17213237

[14] Flöel A Werner C Grittner U Physical fitness training in Subacute Stroke (PHYS-STROKE)--study protocol for a randomised controlled trial. Trials 2014;15:45. 10.1186/1745-6215-15-45 24491065 PMC3922602

[15] Mahoney FI Barthel DW Functional Evaluation: The Barthel Index. Md State Med J 1965;14:61-5. 14258950

[16] Medical University of Graz. Randomizer. www.randomizer.at/. Accessed April 09, 2018.

[17] Bushnell C Bettger JP Cockroft KM Chronic Stroke Outcome Measures for Motor Function Intervention Trials: Expert Panel Recommendations. Circ Cardiovasc Qual Outcomes 2015;8(Suppl 3):S163-9. 10.1161/CIRCOUTCOMES.115.002098 26515205 PMC5289112

[18] Collen FM Wade DT Robb GF Bradshaw CM The Rivermead Mobility Index: a further development of the Rivermead Motor Assessment. Int Disabil Stud 1991;13:50-4. 10.3109/03790799109166684 1836787

[19] Nave AH Kröber JM Brunecker P Biomarkers and perfusion--training-induced changes after stroke (BAPTISe): protocol of an observational study accompanying a randomized controlled trial. BMC Neurol 2013;13:197. 10.1186/1471-2377-13-197 24330706 PMC3870989

[20] Adams HP Jr Davis PH Leira EC Baseline NIH Stroke Scale score strongly predicts outcome after stroke: A report of the Trial of Org 10172 in Acute Stroke Treatment (TOAST). Neurology 1999;53:126-31. 10.1212/WNL.53.1.126 10408548

[21] Wood SN Stable and Efficient Multiple Smoothing Parameter Estimation for Generalized Additive Models. J Am Stat Assoc 2004;99:673-86. 10.1198/016214504000000980 .

[22] IBM. Released 2016. IBM SPSS Statistics for Windows, Version 24.0. Armonk, NY: IBM.

[23] StataCorp Stata Statistical Software: Release 14. StataCorp, 2015.

[24] R Core Team. 2017. R: A language and environment for statistical computing. R Foundation for Statistical Computing, Vienna, Austria. URL www.R-project.org/.

[25] van Buuren S Groothuis-Oudshoorn K mice: Multivariate Imputation by Chained Equations in R. J Stat Softw 2011;45 10.18637/jss.v045.i03

[26] Loudon K Treweek S Sullivan F Donnan P Thorpe KE Zwarenstein M The PRECIS-2 tool: designing trials that are fit for purpose. BMJ 2015;350:h2147. 10.1136/bmj.h2147 25956159

[27] van de Port IGL Wevers LEG Lindeman E Kwakkel G Effects of circuit training as alternative to usual physiotherapy after stroke: randomised controlled trial. BMJ 2012;344:e2672. 10.1136/bmj.e2672 22577186 PMC3349299

[28] English C Bernhardt J Crotty M Esterman A Segal L Hillier S Circuit class therapy or seven-day week therapy for increasing rehabilitation intensity of therapy after stroke (CIRCIT): a randomized controlled trial. Int J Stroke 2015;10:594-602. 10.1111/ijs.12470 25790018

[29] Globas C Becker C Cerny J Chronic stroke survivors benefit from high-intensity aerobic treadmill exercise: a randomized control trial. Neurorehabil Neural Repair 2012;26:85-95. 10.1177/1545968311418675 21885867

[30] Duncan P Studenski S Richards L Randomized clinical trial of therapeutic exercise in subacute stroke. Stroke 2003;34:2173-80. 10.1161/01.STR.0000083699.95351.F2 12920254

[31] Eich H-J Mach H Werner C Hesse S Aerobic treadmill plus Bobath walking training improves walking in subacute stroke: a randomized controlled trial. Clin Rehabil 2004;18:640-51. 10.1191/0269215504cr779oa 15473116

[32] Biernaskie J Chernenko G Corbett D Efficacy of rehabilitative experience declines with time after focal ischemic brain injury. J Neurosci 2004;24:1245-54. 10.1523/JNEUROSCI.3834-03.2004 14762143 PMC6793570

[33] Mehrholz J Thomas S Elsner B Treadmill training and body weight support for walking after stroke. Cochrane Database Syst Rev 2017;8:CD002840. 10.1002/14651858.CD002840.pub4 28815562 PMC6483714

[34] da Cunha IT Jr Lim PA Qureshy H Henson H Monga T Protas EJ Gait outcomes after acute stroke rehabilitation with supported treadmill ambulation training: a randomized controlled pilot study. Arch Phys Med Rehabil 2002;83:1258-65. 10.1053/apmr.2002.34267 12235606

[35] Strømmen AM Christensen T Jensen K Intensive treadmill training in the acute phase after ischemic stroke. Int J Rehabil Res 2016;39:145-52. 10.1097/MRR.0000000000000158 26926203

[36] Winstein CJ Stein J Arena R Guidelines for Adult Stroke Rehabilitation and Recovery: A Guideline for Healthcare Professionals from the American Heart Association/American Stroke Association. Stroke 2016;47:e98-e169 10.1161/STR.0000000000000098 27145936

[37] AVERT Trial Collaboration group Efficacy and safety of very early mobilisation within 24 h of stroke onset (AVERT): a randomised controlled trial. Lancet 2015;386:46-55. 10.1016/S0140-6736(15)60690-0 25892679

[38] Tyson SF Thomas N Vail A Tyrrell P Recruiting to inpatient-based rehabilitation trials: lessons learned. Trials 2015;16:75. 10.1186/s13063-015-0588-2 25886846 PMC4350304

[39] Winters C van Wegen EEH Daffertshofer A Kwakkel G Generalizability of the Maximum Proportional Recovery Rule to Visuospatial Neglect Early Poststroke. Neurorehabil Neural Repair 2017;31:334-42. 10.1177/1545968316680492 27913798

