# Supplementary material for: Physical Fitness Training in Patients with Subacute Stroke (PHYS-STROKE): multicentre, randomised controlled, endpoint blinded trial
Source: BMJ. 2019 Sep 18;366:l5101. doi: 10.1136/bmj.l5101 (PMC6749174; doi:10.1136/bmj.l5101)
Supplement: Supplementary file 1 — Supplementary material: inclusion and exclusion criteria [file nava049481.ww1.pdf]

Inclusion and Exclusion Criteria of the PHYS-STROKE trial.

**INCLUSION CRITERIA**	
**1.**	**Diagnosis of stroke (inclusion within 5-45 days after stroke onset); ischemic or haemorrhagic (cortical, subcortical, brainstem), as determined by initial MRI/CT scan of the brain)**
2.	Age ≥ 18 years
3.	Able to sit unsupported (i.e. without holding onto supports such as the edge of the bed), with feet supported, for at least 30 seconds
4.	Barthel-Index ≤ 65 at inclusion
5.	Considered able to perform aerobic exercise, as determined by responsible physician
6.	Provision of written informed consent
**EXCLUSION CRITERIA**	
1.	Patient considered unable to comply with study requirements
2.	Stroke due to intracranial haemorrhage primarily due to bleeding from ruptured aneurysm or arteriovenous malformation
3.	Progressive stroke
4.	Unable to perform the required exercises due to a) medical, b) musculo-skeletal, or c) neurological problems (for details see below, 4a-c)
4a.	Medical problems: unstable cardiovascular condition, or other serious cardiac conditions (e. g., New York Heart Association criteria for Class IV heart disease, hospitalization for myocardial infarction or heart surgery within 120 days, severe cardiomyopathy or documented serious and unstable cardiac arrhythmias)
4b.	Musculoskeletal problems: restricted passive range of motion in the major lower limb joints (i.e. an extension deficit of > 20° for the affected hip or knee joints, or a dorsiflexion deficit of > 20° for the affected ankle)
4c.	Neurological problems: severity of stroke-related deficits
5.	Required help of at least 1 person to walk before stroke due to neurological (e. g., advanced Parkinson's disease, Amyotrophic Lateral Sclerosis, Multiple Sclerosis) or non-neurological co-morbidities (e. g. heart failure, orthopaedic problems)
6.	Life expectancy of less than 1 year as determined by responsible physician
7.	Drug or alcohol addiction within the last six months
8.	Significant current psychiatric illness defined as medication-refractory of bipolar affective disorder, psychosis, schizophrenia or suicidality.
9.	Current participation in another interventional trial

MRI = magnetic resonance imaging | CT = computer tomography.
