# Supplementary material for: Physical Fitness Training in Patients with Subacute Stroke (PHYS-STROKE): multicentre, randomised controlled, endpoint blinded trial
Source: BMJ. 2019 Sep 18;366:l5101. doi: 10.1136/bmj.l5101 (PMC6749174; doi:10.1136/bmj.l5101)
Supplement: Supplementary file 2 — Supplementary material: appendix [file nava049481.ww2.pdf]

Physical Fitness Training in Patients with Subacute Stroke (PHYS-STROKE) – a multicentre, randomised-controlled, endpoint-blinded trial

Supplementary Appendix

Table of contents

List of investigators of the PHYS-STROKE trial 3

Trial Boards and Committees 4

Steering Committee: 4
Data Safety Monitoring Board: 4
Data Monitoring: 4
Responsible Biostatistician: 4
Trial centre 4
Study assessors: 4

Supplementary Methods 5

Study Design 5
Study protocol 5
Study protocol: Amendments 6
Randomisation and masking 6
Procedures: Care providers 6
Procedures: Intervention manual 6
Procedures: Protocol adherence 10
Procedures: Standard Care 10
Outcomes: Assessments of Secondary Endpoints 10
Statistical Analysis Plan 13
Statistical Analysis Plan: Changes 13
Statistical Analysis Plan: Imputation 14
Data sharing 14

Supplementary Results 14

Protocol adherence 14
Protocol adherence: Target Heart Rate assessment 16
Outcomes: Intervention facilities 17
Outcomes: Imputation 17
Outcomes: Exploratory analysis of subgroups 19
Standard care 20

Supplementary information for patients and carers 21

References 22

List of investigators of the PHYS-STROKE trial

Centre (study period; number of patients enrolled / number of patients randomised)

Center for Stroke Research Berlin (CSB): A Flöel (PI), M Ebinger, AH Nave, T Rackoll, U Grittner (biostatistician), A Meisel, HJ Audebert, S Hesse (deceased).

Charité-Universitätsmedizin Berlin, Institute of Neurology (*15.10.2014 – 21.04.2017; 25 / 25*): A Flöel (PI), M Ebinger, AH Nave, A Meisel, HJ Audebert, F Klostermann.

Charité-Universitätsmedizin Berlin, Evangelisches Geriatriezentrum Berlin (*04.03.2014 – 03.11.2017; 26 / 25*): U Müller-Werdan, E Steinhagen-Thiessen.

Median Klinik Grünheide (*23.10.2013 – 11.10.2017; 19 / 19*): H Bläsing.

Median Klinik Berlin-Kladow: centre closed.

Kliniken Beelitz GmbH, Berlin (*16.10.2013 – 31.07.2017; 33 / 32*): A Gorsler.

Vivantes Klinikum Neukölln, Berlin, Klinik für Neurologie (*08.06.2015 – 08.11.2017; 21 / 21*): D Nabavi.

Medical Park Berlin Humboldtmühle, Berlin (*26.09.2013 – 27.10.2017; 76 / 75*): M Ebinger.

University Medicine Greifswald, Department of Neurology: A Flöel (PI).

Brandenburgklinik Berlin-Brandenburg, Abteilung Neurologie (*27.09.2013 – 08.04.2014; 4 / 3*): centre closed.

Trial Boards and Committees

Steering Committee:

Agnes Flöel (Chair),

Martin Ebinger,

Alexander Heinrich Nave,

Andreas Meisel,

Matthias Endres.

Data Safety Monitoring Board:

Gerhard Jan Jungehülsing (Neurologist),

Diethard Steube (Neurologist).

Data Monitoring:

Alexa Ziegeler,

Karina Sterenberg,

Heidi Schimke.

Responsible Biostatistician:

Ulrike Grittner.

Trial centre

Regina Schlieder,

Torsten Rackoll.

Study assessors:

Daniela Krohne (physiotherapist),

Dike Remstedt (occupational therapist).

Supplementary Methods

Study Design

The study was designed to test the efficacy of an aerobic physical fitness intervention that can be administered in a clinical setting. Thus, trial interventions were directly applied at neurorehabilitation clinics next to usual care. Several aspects of the trial design follow principles of pragmatic trials. Accordingly, we have evaluated the PHYS-STROKE trial regarding the nine dimensions proposed by the Pragmatic–Explanatory Continuum Indicator Summary 2 (PRECIS-2) tool for assessing the level of pragmatism in a trial (1). As a result, PHYS-STROKE should be regarded as a rather pragmatic trial (see Figure S1).

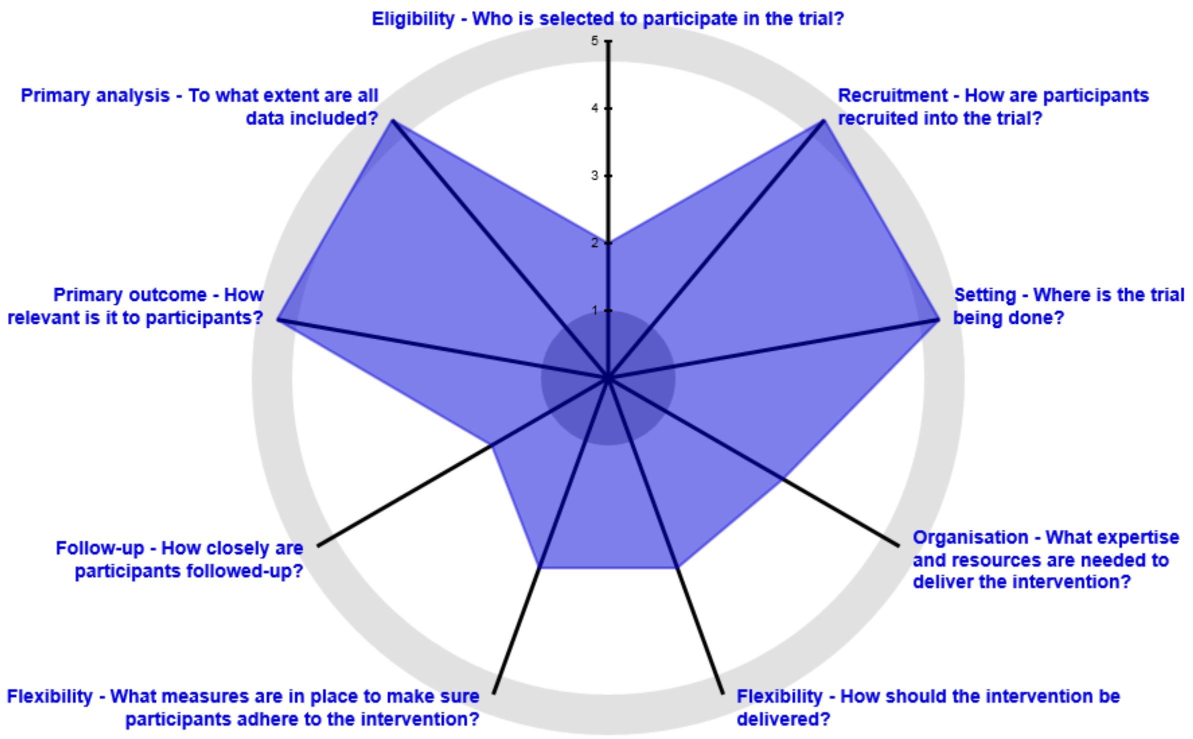

Figure S1: The Pragmatic–Explanatory Continuum Indicator Summary-2 (PRECIS-2) wheel of the PHYS-STROKE trial. Nine dimensions display the level of pragmatism of a trial with scores ranging from one (very explanatory) to five (very pragmatic).

Study protocol

The English version of the study protocol[1] was first published in the 'Trials' Journal:

Flöel A, Werner C, Grittner U, *et al*. *Physical fitness training in Subacute Stroke (PHYS-STROKE)--study protocol for a randomised controlled trial*. Trials 2014; 15: 45.

Study protocol: Amendments

In the course of the study the ethics committee of the Charité-Universitätsmedizin Berlin approved four amendments (02.09.2013; 23.04.2015; 15.11.2016; 21.08.2017). The following data were added:

Table S1: Amendments approved by the ethics committee of the Charité-Universitätsmedizin Berlin.

Amendment and Date of Votum	Changes	Reasons for changes
Amendment and Votum 23.04.2015	Seven day accelerometry,<br>Freiburg questionnaire on physical activity (short version).<br>Implementation of two new trial sites (Charité-Universitätsmedizin Berlin, Institute of Neurology and Vivantes Klinikum Neukölln, Berlin, Klinik für Neurologie).	At follow-up 3 months post stroke, most subjects are still in inpatient clinical care where daily variation of physical activity is limited. Variation of physical activity is greater in a home setting. Thus, a seven-day accelerometry is considered a more precise assessment at follow-up 6 months post stroke.<br>The German Freiburg questionnaire on physical activity is validated in a broad age range in the German population and is capable of estimating daily energy expenditure. It is regarded as a valuable measure in addition to the accelerometry measurement to assess long-term effects of the intervention on physical activity.<br>Two more trial sites are added as a consequence of difficulties in recruitment.
Amendment and Votum 15.11.2016	Audio recording of the Regensburg semantic and phonemic word fluency test.	To increase the explanatory power of the Regensburg semantic and phonemic word fluency test, audio recordings are necessary to distinguish temporal patterns (temporal cluster analysis) of word production.
Amendment and Votum 12.09.2017	Small administrative changes (no additional assessments).	The local PI changed from Dr. Flöel to Dr. Endres due to a change of the affiliation of Dr. Flöel.

Randomisation and masking

Randomisation used Functional Ambulation Category (FAC; dichotomised FAC≤3), centre, and age (dichotomised age at ≤65 years) as strata and was done in a 1:1 fashion. Each strata was organised in blocks of 10 subjects. The randomisation procedure was done by a clinician of each study site after the baseline visit was performed. The assignment was subsequently communicated to the treating therapists. The trial centre was not informed about patient allocation during the course of the trial.

Procedures: Care providers

Each study site assigned at least two therapists per intervention group to the trial, who were responsible for conducting the intervention. Care providers were supposed to be physiotherapists or sport therapists by training delivering the PHYS intervention and neuropsychologists or psychologists for the RELAX intervention respectively. A team member of the trial centre regularly visited each study site to discuss issues regarding the intervention and to control the adherence to adequate documentation. In addition, the trial monitoring controlled for every patient whether intervention documentation was adequate.

Procedures: Intervention manual

A written intervention manual was distributed to all trial therapists containing extensive description of the intervention and documentation procedures. Additionally, a web page with frequently asked questions was

maintained. A regular newsletter informed about specific procedures. A member of the trial centre visited each study site to discuss current issues with the treating therapists. The intervention manuals for both, PHYS and RELAX, are described in the following section:

PHYS – Intervention (experimental)

The aerobic physical fitness training (PHYS) intervention was designed to improve cardiorespiratory fitness while being functional in terms of applying locomotion therapy. The goal was to reduce deconditioning and enhance endogenous neuroplasticity following a cerebrovascular event. Cardiorespiratory load was targeted at 50–60 % of the estimated maximal heart rate to ensure an aerobic training in the lower range of recommended cardiorespiratory training intensities endorsed by the American College of Sports Medicine (ACSM) (2).

The target heart rate (THR) was calculated by care providers (therapists) at the beginning of the intervention period based on the formula: '180 – age'. The THR was adjusted with minus 10 beats in case of beta blocker intake. Before the first intervention session patients were allocated to receive training either in a gait trainer (FAC score 0 – 2) or on a body-weight supported treadmill (FAC 3 – 5). FAC was assessed by the therapists on a daily basis and subsequent training was administered using the respective allocated device.

Before each session, resting (pre-training) heart rate (HR) and blood pressure (RR) was assessed while the patient was still in a resting position. Training was only commenced if HR was below 180 bpm or systolic blood pressure below 200 mmHg, as recommended by the ACSM. Additionally, patients were equipped with a pulse belt for the therapist to constantly monitor the patient's HR.

Gait trainer:

The intervention manual refers to the Gait Trainer GT1, Reha-Stim, Berlin, Germany. General recommendations equally applied to other manufacturers. The patient was seated in the gait trainer following the manufacturer's manual. Attention was paid to the individual step length, and ensured that the hemiparetic arm was fixed to the handle bar in front of the patient. Body-weight support (BWS) was adjusted by the therapist, and only given as functionally needed. Training started with a three minute warm-up phase in which the patient started to practice at a low speed. After three minutes, the speed was increased until the THR was reached. After twenty minutes, the speed was reduced for another two minutes to reach a cool-down phase. Training at the THR was possible and constantly achieved due to the following adjustments of treatment modalities: decreasing BWS or increasing speed. The training aimed at delivering a constant cardiorespiratory load of 50 – 60 % maximum HR, represented by the THR. In addition patients who recover functionally were able to transition to treadmill based training if FAC was > 2.

Treadmill:

The intervention manual refers to the Multi-disk treadmill Callis, Model Therapie, SPRINTEX Trainingsgeräte GmbH, Kleines Wiesental, Germany. General recommendations equally applied to other manufacturers. The patient was supplied with the BWS irrespective of need to ensure safety. Body-weight support (BWS) was adjusted by the therapist only as much as functionally needed to allow proper trunk and limb alignment as well as weight shifting on the paretic limb. Patients started at the preferred walking speed over a warm-up period of three minutes. After three minutes, the speed was increased until the THR was reached. After twenty minutes of training, the speed was reduced for another two minutes to the individually preferred walking speed to reach a cool-down phase. Training at the THR was possible and constantly achieved due to the following adjustments of treatment modalities: increase of inclination, reduction of BWS, and increase of speed. The goal was to reduce BWS until 0 % was reached while still allowing for a proper weight bearing of the paretic limb with less than 15° of knee flexion. Patients were able to use handle bars, if needed. Therapists were advised to provide functional support by facilitating hip movement, support weight shifting, stabilizing of the knee, or setting of the paretic limb in case of paresis of the peroneus muscles. Recommendations were given for positioning of staff to ensure ergonomic posture of the therapists during foot placement assistance. Patients were allowed to use orthoses if used also during usual care physiotherapy.

After the training the subject was seated on a chair to assess post-training HR, RR, and perceived rate of exertion during training on a visual analogue scale (0 – 10; 10 denotes highest possible exertion). After all equipment had been removed from the patient, he or she was asked if any adverse events occurred during the last 24 hours.

Safety procedures:

Throughout the training, HR was monitored by the treating therapist. In case of a HR increase above 180 bpm training was stopped and a physician was contacted. Additionally training was stopped if the patient reported strong pain, constant dizziness, severe fatigue, or a strong urge to urinate. Patients were allowed to take short breaks, but therapists were advised to resume training as quickly as possible. The number and length of breaks were to be decreased over time. To ensure the patient was still practicing below the anaerobic threshold the talk test (3) was administered during each intervention session.

RELAX – Intervention (control)

The relaxation control intervention was designed according to the muscle relaxation after Jacobsen (4) in order to release from overall stress but to restrain from cardiorespiratory load.

A quiet room with either a comfortable chair or bed was used to administer the intervention. Before each session resting heart rate (HR) and blood pressure (RR) was assessed while the patient was still in a resting position. Additionally the patient was equipped with a pulse belt to constantly monitor HR. The patient was positioned to enable relaxation, and soft cushion support was provide to the limbs if needed.

The care provider (therapist) read the commands slowly to the patient (see Table S2). Additionally the patient was encouraged to focus on the feeling of warmth and heaviness in the addressed muscle group. Throughout the intervention session, the therapist ensured that the patient focused on the relaxation, but did not fall asleep.

After 25 minutes of relaxation training, HR and RR were documented and perceived rate of exertion assessed on a visual analogue scale (0 – 10; 10 denotes highest possible exertion). After all equipment had been removed from the patient, he or she was asked if any adverse events occurred during the last 24 hours.

The RELAX intervention was stopped if patients reported severe pain, strong fatigue, or the urge to urinate, and continued after the problem had been solved.

Table S2: Routines for relaxation program.

No.	Phase	Instruction
1 Introduction		
1.1	R	Close your eyes, take several deep breaths, relax and feel how your body becomes loose and heavy. Try to not think about anything. Sense all the muscles in your body and try to relax as well as possible.
2 Hands and arms		
2.1	T	Now focus on your right hand. Make a fist and observe the tension. Hold your fist and feel the tension within your fist and the forearm.
2.2	R	And now relax. Let the fingers of your right hand go loose and pay attention to the difference.
2.3	T	And now do the same with your left hand. Make a fist while your body is relaxing and concentrate on the tension.
2.4	R	And now relax…
2.5	T	Clench now both fists and pay attention to your sensations.
2.6	R	And now release. Stretch your fingers and feel the relaxation. Progress in letting your hands and forearms go loose … Your hands are now warm and heavy.
3 Upper arm		
3.1	T	Now bend your elbows and strain your biceps. Pay attention on the sensation of tension.
3.2	R	And now stretch your arms again and focus on the difference. Notice how relaxation starts to spread.
3.3	T	Now extend your arms, and push them on the pad so that you have a strong sensation of tension in your upper arm. Feel the tension.
3.4	R	And now relax. Place your arms comfortably. Let the sensation of release spread. You feel a strong sensation of heaviness in your arms, while they relax.
4 Forehead and eyes		
4.1	T	Now pull your eyebrows towards your forehead so you feel wrinkles form on your forehead. Hold the tension.
4.2	R	And now relax your forehead and let it go loose and smooth again. Pay attention on how your skin becomes softer the more you relax. The entire skin relaxes.
4.3	T	Now pull your eyebrows together, so that a vertical wrinkle appears between your eyes. Pay attention to the sensation of tension.
4.4	R	And now relax again. Let your forehead go loose.
4.5	T	Squint your eyes tightly … and feel the tension
4.6	R	And now relax again. Let your eyes and your cheeks go loose and pay attention on the relaxation
5 Jaw muscles and lips		
5.1	T	Now push your teeth together. Pay attention to the tension which evolves in your jaw muscles.
5.2	R	Relax your jaw muscles. Leave your lips open just a little bit. Feel yourself relax.
5.3	T	Now tightly press your tongue against the roof of the mouth. Focus on the tension.
5.4	R	Release your tongue again and relax.
6 Throat		
6.1	T	Now turn your attention towards your neck muscles. Push the back of your head smoothly into the pad. While doing so your chin moves towards the breast bone. Tense your throat and neck muscles and focus on the tension.
6.2	R	And now release your neck. Place your head again in a comfortable position.
6.3	T	Bend your head to the front and push your chin against your chest as well as possible. And experience the sensation of tension.
6.4	R	Now place your head on the pad again and focus on the sensation of relaxation. Let the relaxation spread.
7 Neck and shoulders		
7.1	T	Now pull your shoulders towards your ears. Hold the tension.
7.2	R	Release your shoulders and experience how the neck and shoulders relax. Pay attention on how well all muscles release tension.
8 Shoulder blades		
8.1	T	Now pull your shoulder blades together and towards the back. Focus on how you experience the feeling of tension and where it is strongest.
8.2	R	And now let your shoulder blades return to the normal position and relax. Let the relaxation spread from your shoulders all the way towards the muscles of your back. Relax your neck, the throat, your jaw muscles and your entire face. Feel how a deep sensation of relief is spreading.
9 Chest		
9.1	R	Breath easy in and out. Pay attention on how your relaxation evolves with you breathing out. And while you exhale you feel the relief.
9.2	T	Now take a deep and strong breath and let it fill your lungs. Hold your breath for a short time. Pay attention to the sensation of tension.
9.3	R	... and let the air flow out by itself. Let your chest release. Feel the relief and continue to breath normally

Tension phase of muscles are supposed to be hold for 5 – 10 seconds while relaxation phase is supposed to yield for about 30 – 40 seconds. Only exception is for the chest in which the tension phase is much longer. T denotes tension, R denotes relaxation.

If in any of the two intervention arms, a session was missed for any reason, study sites were asked to administer missed sessions until twenty sessions were reached. Patients were not allowed to miss training on more than five consecutive days.

Procedures: Protocol adherence

To achieve a standardised intervention regimen, therapists of study centres were trained in the application of the intervention procedures (instructor: Daniela Krohne) and in the documentation of daily intervention diaries following GCP-guidelines (instructor: Regina Schlieder). Quality checks of the intervention diaries were done with every monitoring visit. Additionally, regular visits were conducted at study sites by a member of the trial coordinating centre (Torsten Rackoll) to discuss the enrolment progress and answer inquiries concerning the intervention. Intervention diaries of both groups documented the time of attention of therapists, time spent in the active phase of each session (core intervention), heart rate, and blood pressure before and after the intervention as well as ratings of perceived exertion and adverse events that occurred in the course of the last 24 hours.

Procedures: Standard Care

Standard care was delivered following the German guidelines for neurorehabilitation after stroke (www.bar-frankfurt.de). Neurorehabiliation in Germany is categorized into several phases depending on the medical status of the patient and is organized as follows:

Phase B: Early rehabilitation (Barthel-Index < 30 points or need of acute medical treatment)

Phase C: Continuing rehabilitation (Barthel-Index 30 – 65)

Phase D: Rehabilitation following inpatient treatment (Barthel-Index 65 – 100)

Besides activating nursing, standard of care in German neurorehabilitation centres consists of physiotherapy, occupational therapy, physical therapy, neuropsychological therapy, speech and facio-oral therapy (http://www.icd-code.de/ops/code/8-552.html). In patients with a BI below 30 points, 300 minutes of therapy sessions per day are standard. In patients with a BI >65 points, the administered therapy time depends on its type: For physiotherapy, at least 180 minutes per week are provided (https://www.deutsche-rentenversicherung.de/Allgemein/de/Inhalt/3_Infos_fuer_Experten/01_sozialmedizin_forschung/downloads/quali_rehatherapiestandards/Schlaganfall/rts_schlaganfall_download.html). In patients with a BI between 30 and 65, the therapy duration may vary at the discretion of the treating physician. Content of each therapeutic approach are defined by the treating therapist and may vary in between study centres.

Recommendations follow the guidelines of the American Heart Association Stroke Council (13). To assure equal administration of therapies we assessed the duration of therapies received per patient between baseline and follow-up three months after stroke. The length of therapy sessions per patient was recorded by the study site during the period of inpatient rehabilitation therapy. After discharge from the study site, patients documented subsequent outpatient rehabilitation therapies in minutes and presented all documentation at follow-up visits.

Outcomes: Assessments of Secondary Endpoints

All outcome measures were assessed using a standardised manual. Study assessors trained the ratings until they reached an agreement for all ratings in each assessment.

6-min Walking Test (6-MWT):

The distance walked in six minutes (6-minute Walking Test, 6-MWT) was assessed at each centre using hallways not used by other patients or personnel during the test. Thirty-five metres were marked with a clear start and ending mark. The 6-MWT was conducted after the 10-metre gait test, but with a resting period in between. Subjects were asked to walk in a speed in which they would be able to walk safely for six minutes without interruption, but to try to cover as much distance as possible. Patients used the same orthoses and / or walking aids during walking

which they used during standard rehabilitative care. An additional person from the assessment team secured every patient's safety and assisted with walking, if needed. The test commenced when subjects started to walk and ended after six minutes. Patients were instructed to turn after 35 metres. The distance walked after six minutes was marked by the assessor. Blood pressure was measured directly after the test and exhaustion was subsequently assessed using a visual analogue scale (zero [0] marking the least possible effort and ten [10] the maximal exhaustion).

Rivermead Mobility Index (RMI) and Rivermead Motor Assessment Subtest Arm:

The Rivermead Mobility Index assesses functional mobility with 15 tasks in increasing difficulty. Patients were instructed to perform each task starting with the least challenging one. Each patient was allowed three attempts per task. If a patient was not able to perform one test, he or she was allowed to try the next, more challenging one. The assessor gave precise instructions and demonstrated the task, if instructions were not understood correctly. The last correctly finished task was counted as the maximum score.

Rivermead Motor Assessment Subtest arm is a subscale ("Upper Limb/Extremity" ('Arm')) of the Rivermead Motor Assessment that determines motor performance of patients after stroke, and consists of 15 arm movements such as pronating/supinating the forearm, bouncing a ball as well as functional items such as cutting putty, grasping and releasing objects, and tying a bow. It consists of test items in three sections that are ordered hierarchically, i.e., first items are easier and become more difficult towards the end of the evaluation. The subtest Arm is assessed as described above for the RMI.

Modified Ranking Scale (mRS):

Trained assessors received information of functional parameters from inpatient care centres and performed all ratings.

Actigraphy:

GT3x accelerometers were initialized using ActiLife Software, Version 6.8.2 (Actigraph Corp, Pensacola, USA) with 100 Hz sample rate and programmed to record the entire day (24 hrs. starting at 12 a.m.) starting the day after each study visit. The assessors explained the rationale of the devices and asked the patient to wear the device until the morning after the recording started. Patients were requested to wear the device the entire time and only take them off during washing or because of extreme discomfort. In order to guarantee a correct relocation of the device in case it was taken off, patients were shown how to place the device by the assessor. If patients suffered from severe paresis of the upper limb, personnel or relatives were instructed to handle the device. The actigraph was placed on the paretic ankle joint with the device pointing to the lateral side. For return shipping patients were provided with prepaid envelopes. Data were downloaded in 60-second intervals. Wear time validation as well as data scoring for step count assessment were executed using ActiLife software. As a cut-off point, we used more than 60 minutes of continuous zeros, with allowance of 1–2 minutes with counts between 1 and 100. Rated non-wear times were subsequently removed from the analyses. For step count, we applied the company-made low frequency extension filter to discriminate steps of slow walkers from random noise, as suggested by Webber & St. John.[3] As a cut-off we used the filter for patients exhibiting a walking speed ≤0.4 m/s.

Walking aids

During gait assessments (10m Walk West and 6-minute Walk Test) patients used the same walking aids and/or orthoses as during standard care physio therapy. All walking aids and orthoses were noted by trial assessors. For statistical analysis walking aids were dichotomised as follows.

0: no walking aid.

1: walking aid (walking frame, walking can, four point walking cane).

Box and Block Test:

The Box and Block Test (BBT) (5) assesses gross manual dexterity of the upper limb to determine functional levels of the upper extremity in people with disability compared with those without disability. The test consists of two wooden boxes and a partition in the middle. One of the boxes is filled with 150 squared blocks with a length of the edges of 2.5 cm each. The patient is seated in front of the boxes. After a precise description of the task and verification that the task was understood correctly, the patient is asked to move as many blocks as possible over the partition. He or she is given 60 seconds per hand. After the 60 seconds the examiner counts the number of blocks the patient has moved. If the patient has moved two or more blocks at the same time those blocks are subtracted from the result.

Medical Research Council Scale:

The Medical Research Council (MRC) (6) scale is the accepted clinical tool for assessing muscle strength. It is rated on an ordinal scale ranging from 0 (plegic) to 5 (full strength), that has shown high intra- and inter-rater reliability. We evaluated the muscle force of six functional muscle groups (hip extension and flexion, knee extension and flexion as well as ankle extension and flexion) of the lower limb.

Trained study assessors compared the impaired with the non-impaired leg assessed the muscle strength. The force of the impaired leg is noted. Prior to the trial, all assessors validated their ratings against each other and against the trainer.

Resistance to passive movement scale:

The Resistance to passive movement scale (REPAS) (7) is a scale measuring spasticity in the lower and upper limbs, comprising eight muscle groups per side in the upper limb, five muscle groups per side in the lower limb, and one overall sum score. It is based on the Ashworth and the modified Ashworth scale, the most commonly used measures for spasticity/resistance to passive movement. The REPAS manual provides instructions for both test administration and scoring of various passive limb motions, showing high internal consistency and reliability for the clinical assessment of resistance to passive movement in patients with upper motor neuron paresis.

Within each muscle group spasticity is rated with a 4-point scale (0 denotes ‘no increase in muscle tone’, 4 denotes ‘fixed in extension and flexion’). The patient is positioned on a bed, and asked to relax the assessed limb as much as possible. The assessor moves each limb starting slowly in the beginning. If neither spasticity nor pain is experienced the limb is moved more quickly to measure finer grades of spasticity.

Center for epidemiologic studies depression scale (CES-D):

The CES-D (8) is a 20-item measure in which patients rate how often they experienced symptoms associated with depression. Response options range from 0 to 3 for each item (0 = Rarely or None of the Time, 1 = Some or Little of the Time, 2 = Moderately or Much of the time, 3 = Most or Almost All the Time). Scores range from 0 to 60, with high scores indicating greater depressive symptoms. The questionnaire is handed to the patients and filled according to the official manual at each visit. Only in cases of severe paresis of the dominant hand or visual impairment the questionnaire is read to the patient and answers documented by the assessor.

The CES-D provides with a lie criteria which rates validity of the answers provided. We used -28 as a cut off and consequently excluded patients with a value below the cut off from analysis.

EuroQol quality of life questionnaire (EQ-5D-5L):

The EQ-5D-5L is a questionnaire in which the patients rate (1 = no problem – 5 = severe problem) their wellbeing within five dimensions as well as rate the impression of one’s health on a vertical visual analogue scale. Questionnaires were filled by each patient at each visit except from those with paresis in their dominant hand. Calculations of EQ-5D-5L was done using the German validation set recently published.(9)

Pittsburgh Sleep Quality Index (PSQI):

The PSQI (10) is a measure to rate sleep and asks for reasons for bad sleep. It is filled at each visit. Except for baseline the PSQI asks for sleep behavior during the last four weeks preceding the visit. At baseline the PSQI is filled with respect to the sleep behavior within the four weeks prior to the cerebrovascular event.

Montreal Cognitive Assessment (MOCA):

The MOCA (11) is a screening tool for cognitive impairment and has a good validity in the stroke population and it provides with three different versions. It is filled at each visit. Except for the six months follow-up visit a different version is used at each assessment. At the six months follow-up visit the version from the baseline visit is filled.

Trail Making Test (TMT):

The trail making test (12) is a neuropsychological test which assesses executive functioning and task switching. The test is administered to each patient at each visit. Cases with an initial neglect symptom were excluded from analysis as the neglect was most likely to overshadow the performance in executive functioning.

Regensburger Wortflüssigkeitstest (RWT; word fluency):

The RWT asks for phonemic and semantic word fluency in four tasks. The sum score is equivalent to the words produced within each minute per tasks minus the mistakes. The test is only administered at baseline and at the three months follow-up visit.

Statistical Analysis Plan

A brief description of planned statistical analyses were published in the study protocol (14). The detailed statistical analysis plan (SAP) was published prior to unblinding (SAP published: Sept 1, 2017; last patient's follow-up visit: Nov 1, 2017) and can be found here:

https://doi.org/10.6084/m9.figshare.5375026.v1

Statistical Analysis Plan: Changes

Handling of missing values

- In the analysis plan from the study protocol published in Trials (14) it was stated to use baseline value imputation in case of missing values.

In the SAP and in the final analyses, we used multiple imputation methods (MICE: multiple imputation by chained equations) for all missing data, except for data missing not at random (MNAR). This approach is regarded as the most appropriate method for missing value imputation in clinical trials (15).

Analyses of safety outcomes

- In the SAP, it was stated to use Fisher's exact test when analysing safety outcomes.

In the primary analyses we used Poisson regression models instead to calculate incidence rates and incidence rate ratios. These models allow accounting for the time at risk for each patient, which differed in case of individual early termination of the trial.

Statistical Analysis Plan: Imputation

Taking into account that our target population was moderately to severely affected, we expected missing values. Handling of missing values and definitions of missings are described here (15,16):

Table S3: Handling of missing data.

Missing data	Handling of missing data
Missing at random (MAR)	Occurrence was expected for follow-up timepoints. We imputed missing values with multivariate imputation by chained equations (17). Imputation was planned to be conducted using the 'miceadds' package of R Statistical Software. Dependent on the variable with missing values groupwise imputation was conducted using that variable with baseline and follow-up data, and additionally the variables centre, sex, age, baseline FAC, and treatment received. We imputed data on the basis of 10 datasets using the r-command:<br><br>mice(dat_for_imp, m = 10, seed = 123)<br><br>With default settings where 'dat_for_imp' denotes the selected variables. The seed was set at '123' to ensure data analysis reproducibility.
Missing completely at random (MCAR)	Occurrence was expected for logistic reasons or failure of measurement equipment. Thus no observed data was at hand to explain missings. Missings termed MCAR were treated in the same way as described for MAR.
Missing not at random (MNAR)	Occurrence was expected for patients unable to be assessed for the reason of general or specific impairment. For baseline we imputed missing values with single value imputation (18). If patients were not able to conduct an assessment due to their impairment as was seen in walking related assessments, single value imputation was used using half of the lowest value observed in the entire cohort.<br>We decided to take the half of the lowest value of the group for patients that were not able to perform the desired task (MNAR), so that the mean of imputed values is lower than the mean of the rest of the cohort. This simple approach generates a very small individual maximal walking speed reflecting the moderately to severely impaired cohort without defining zero as a maximum walking speed at baseline, or loosing data points.

Data sharing

All scripts for the primary endpoint analyses as well as the corresponding datasets can be found here: http://doi.org/10.5281/zenodo.3341240

Supplementary Results

Protocol adherence

At one study site (Charité, Campus Benjamin Franklin) physiotherapists delivered all study interventions (PHYS and RELAX). In all other participating study centres, care providers were available on site delivering the respective study interventions (physiotherapists or sport therapists for PHYS; psychologists and neuropsychologists for RELAX).

Table S4: Additional baseline characteristics

**Characteristic**	**PHYS-Group (n=105)**	**RELAX-Group (n=95)**	**All Patients (n=200)**
Comorbidities			
Atrial Fibrillation, n (%)	23 (22)	23 (24)	46 (23)

Diabetes mellitus, n (%)	32 (31)	31 (33)	63 (32)
Hypertension, n (%)	86 (82)	80 (84)	166 (83)
Hypercholesterolemia, n (%)	43 (41)	37 (39)	80 (40)
Coronary artery disease, n (%)	11 (11)	18 (19)	29 (15)
Tumor, n (%)	12 (11)	8 (8)	20 (10)
Medication			
Antiplatelets, n (%)	70 (67)	69 (73)	139 (70)
Oral anticoagulation, n (%)	20 (19)	20 (21)	40 (20)
Beta blocker, n (%)	49 (47)	47 (50)	96 (48)
Diuretics, n (%)	46 (44)	37 (39)	83 (42)
Calcium channel blockers, n (%)	41 (39)	38 (40)	79 (40)
Statins, n (%)	79 (75)	80 (84)	159 (80)
Antibiotics, n (%)	3 (3)	2 (2)	5 (3)
Analgetics, n (%)	13 (12)	18 (19)	31 (16)
Smoking‖			
Never smoked, n (%)	47 (53)	49 (57)	96 (55)
1-20 pack years, n (%)	19 (21)	14 (16)	33 (19)
>20 pack years, n (%)	23 (26)	23 (27)	46 (26)
Body Mass Index, mean (SD), kg/m^2	26 (4)	26 (4)	26 (4)

‖ History of smoking was not available in 25 patients.

Table S5: Parameters of protocol adherence per intervention group.

**Intervention diaries**	**PHYS**<br>**N = 105**	**RELAX**<br>**N = 95**
Time between baseline visit and intervention start in days, mean (SD)	4 (3)	4 (3)
Number of performed intervention sessions, mean (SD)	16 (6)	17 (5)
Time of attention by therapist per session, mean (SD)	44 (9)	43 (7)
Duration of core intervention in minutes, mean (SD)	21 (4)	24 (3)
Heart rate pre-session in bpm, mean (SD)	78 (10)	73 (10)

Heart rate post-session in bpm, mean (SD)	94 (14)	71 (10)
Heart rate delta in bpm, mean (SD)	15 (9)	-2 (3)
Blood pressure pre-session in mmHg, mean (SD)	126/75 (11/9)	126/74 (12/8)
Blood pressure post-session in mmHg, mean (SD)	128/76 (12/9)	123/73 (12/8)
Visual analogue scale of perceived exertion, mean (SD)*	5 (0)	2 (0)
Reason for stopping intervention sessions§, mean (SD)		
Pain	0 (1)	0 (0)
Urge to urinate	0 (0)	0 (0)
Time	0 (0)	0 (0)
Fatigue	2 (4)	0 (0)
Refusal	0 (0)	0 (0)
Other	0 (1)	0 (0)

* Visual analogue scale from 0 to 10 where 0 stands for no perceived exertion and 10 for maximum perceived exertion. § Displayed are the mean (SD) number of times an intervention session was stopped for any of the listed reasons.

Table S6: Progression in training modalities over time.

	Day 1	Day 20
Distance in metres, mean (SD)	446 (332)	967 (540)
Speed in km/h, mean (SD)	1.6 (1.3)	2.4 (1.1)
Incline used on treadmill in %, mean (SD)	0.4 (1.0)	1.8 (2.1)

We performed a sensitivity analysis to compare adherence to PHYS intervention regimen in severely impaired patients (FAC 0 – 1) to the rest of the training cohort (FAC 2 – 5):

Tables S7: Sensitivity analysis comparing training responses between severely impaired patients to the rest.

	FAC 0 – 1 (n = 41)	FAC 2 – 5 (n = 63)
Sessions, mean (SD)	16 (7)	16 (6)
No. of stopping intervention due to fatigue, mean (SD)	3 (4)	2 (3)
Minutes per session, mean (SD)	21 (5)	21 (4)
Visual analogue scale, mean (SD)	6 (2)	5 (2)
Delta heart rate, mean (SD)	15 (9)	15 (9)

Protocol adherence: Target Heart Rate assessment

We compared the pragmatic PHYS-STROKE approach to estimate the desired target heart rate (THR) of 50 – 60% of a patient's maximum heart rate (maxHR) to a conventional approach from the American College of Sports Medicine (19) which was also used in previous stroke trials (20) calculating 55% of an estimated maxHR (see Table S7). The data presented in Table S8 demonstrate that the PHYS-STROKE approach resulted in a higher desired THR compared to the conventional approach aiming for 55% of an estimated maxHR. This difference was observed throughout all age groups, but was particularly pronounced in younger individuals. The mean age of patients of the PHYS group was 69 (12) years. Therefore, it can be assumed that sufficient cardiovascular stress was induced in patients of PHYS-STROKE allocated to aerobic physical fitness training.

Table S8: Comparison of two formulas to calculate a patient's target heart rate (50-60% of maximum heart rate).

Approach type, in bpm	40 years old	50 years old	60 years old	70 years old	80 years old	90 years old
PHYS-STROKE approach (THR= 180-age)	140	130	120	110	100	90
Conventional approach (55% of maxHR)	99	95	91	87	83	79

The pragmatic PHYS-STROKE approach calculated the target heart rate (THR) with the formula: THR = 180 – age. The conventional approach calculated 55% of an estimated maximum heart rate (maxHR) with the formula: THR = 0.55*(207 – 0.7 * age).

Outcomes: Intervention facilities

Four out of seven study sites had a gait trainer available. We performed a sensitivity analyses on severely affected patients (FAC < 3) who were not trained on a gait trainer as described in the protocol but no difference to the primary outcomes were observed.

Outcomes: Imputation

Data needed to be imputed for missing values due to attrition or calculated for patients too severely affected to complete the assessment of gait. Data imputation displayed is for co-primary and key secondary outcomes.

Table S9: Imputation of missing data.

Reason	All	PHYS	RELAX
Multiple imputation for gait speed of patients not available at follow-up visit 3 months post stroke, no (%). Assuming missing at random MAR.	34 (17%)	16 (15%)	18 (19%)
Calculated data for gait speed of patients not able to walk ten metres at baseline by using half of the lowest value of the cohort, no (%). Assuming missing not at random MNAR.	21 (11%)	9 (9%)	12 (13%)
Multiple imputation for Barthel-Index due to missing values of patients at follow-up visit 3 months post stroke, no (%)∫. Assuming MAR.	32 (16%)	15 (14%)	17 (18%)

∫Two patients (1 PHYS, 1 RELAX) were not present at follow-up visit 3 months post stroke, but the assessment of the Barthel-Index was possible via telephone.

We performed a sensitivity analysis to assess the quality of our multiple imputation approach by giving the imputation a hierarchical order as this has been suggested previously (21). We used centre as the highest order, to impute missing data in a groupwise fashion. However, no improved assumption to missing data was found using hierarchical order as outlined in Table S9. Although it seems reasonable to cluster data in a hierarchical order, in our case at least, centre did not improve the outcome, most likely because of the low number of data in each centre.

Table S10: Sensitivity analysis of co-primary endpoints with hierarchical imputation.

Variable	PHYS (n=105)	RELAX (n=95)	Treatment effect / OR (95% CI)	P Value
**Co-Primary Outcomes**				
Change in maximal walking speed, mean (95% CI), in m/s	0.4 (0.2 to 0.6)	0.3 (0.0 to 0.6)	0.1 (-0.1 to 0.3)	0.46
Change in Barthel-Index, mean (95% CI)	32 (25 to 39)	30 (19 to 41)	0 (-12 to 12)	0.97

Outcomes: Exploratory analysis of subgroups

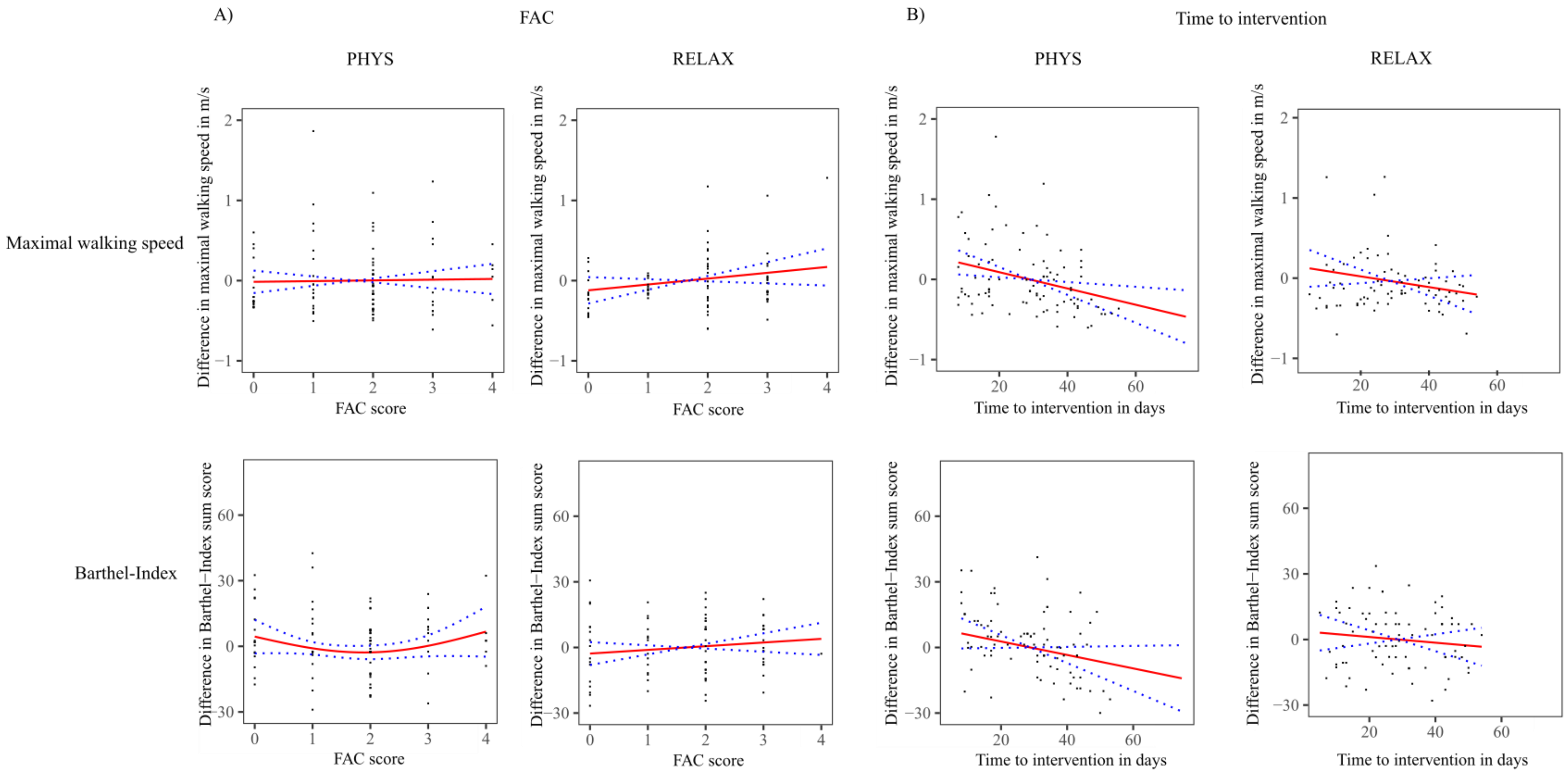

Figure S2: Subgroup analyses demonstrating the difference in maximal walking speed and Barthel-Index (follow up three months after stroke - baseline) as a function of Functional Ambulation Category (FAC, panel A) and the time from stroke onset to start of intervention (Time to intervention, panel B), respectively, using splines.

Standard care

Times of standard care therapies depend on the degree of functional disability. Data have thus a skewed distribution.

Table S11: Inpatient and outpatient therapy times between baseline and follow-up 3 months post stroke and during intervention period only.

**Observation time: Baseline - 3 months post stroke**	**PHYS n = 105**	**RELAX n = 95**	**ALL n = 200**
Physiotherapy, median [IQR], min¶	2220 [1545 – 2782]	2122 [1540 – 2692]	2160 [1530 – 2760]
Occupational therapy, median [IQR], min	1860 [1155 – 2355]	1560 [1140 – 2145]	1680 [1140 – 2284]
Speech therapy, median [IQR], min	960 [600 – 1335]	915 [570 – 1331]	930 [570 – 1335]
Neuropsychological therapy, median [IQR], min	690 [428 – 1008]	600 [480 – 840]	670 [450 – 900]
**Observation time: Intervention period**			
Physiotherapy, median [IQR], min¶	1320 [945 – 1680]	1230 [840 – 1733]	1268 [895 – 1710]
Occupational therapy, median [IQR], min	1050 [600 – 1350]	960 [583 – 1350]	1043 [599 – 1350]
Speech therapy, median [IQR], min	450 [210 – 750]	420 [188 – 655]	450 [210 – 825]
Neuropsychological therapy, median [IQR], min	380 [225 – 510]	360 [210 – 450]	360 [210 – 480]

Data comprise the total of applied inpatient and outpatient therapies in minutes from baseline until follow-up visit three months after stroke. Data of 55 patients were not provided by the study sites.

¶Physical therapy comprises either conventional Bobath therapy or repetitive locomotion therapy depending on the trial site's standard care protocols. Statistical analyses are adjusted for study sites.

Supplementary information for patients and carers

Definitions for acute, subacute and chronic phases are defined as follows and are in line with definitions endorsed by the Stroke Recovery and Rehabilitation Roundtable (SRRR) (22):
Hyperacute: <24 hours post stroke
Acute: 1 – 7 days post stroke
Early Subacute: 1 week – 3 months post stroke
Late Subacute: 3 months – 6 months post stroke
Chronic: > 6 months post stroke

Physical exercise recommendations after stroke:
https://www.ahajournals.org/doi/full/10.1161/STR.0000000000000022

Rehabilitation recommendations:
https://www.nice.org.uk/guidance/cg162/resources/cg162-stroke-rehabilitation-full-guideline3

Standard care physiotherapy after stroke:

https://www.stroke.org.uk/resources/physiotherapy-after-stroke

Barthel-Index:
http://www.strokecenter.org/wp-content/uploads/2011/08/barthel.pdf

Bobath approach:
https://www.physio-pedia.com/Bobath_Approach (English)
https://de.wikipedia.org/wiki/Bobath-Konzept (German)

References

1. Loudon K, Treweek S, Sullivan F, Donnan P, Thorpe KE, Zwarenstein M. The PRECIS-2 tool: Designing trials that are fit for purpose. BMJ. 2015;350.
2. Pescatello LS, Arena R, Riebe D, Thompson PD. ACSM's Guidelines for Exercise Testing and Prescription. Vol. 27, American College of Sports Medicine. 2014. 1-715 p.
3. Reed JL, Pipe AL. The talk test: A useful tool for prescribing and monitoring exercise intensity. Curr Opin Cardiol. 2014 Sep;29(5):475–80.
4. Jacobson E. Progressive relaxation-A physiological and clinical investigation of muscular states and their significance in psychology and medical practice. Jama Network. 1938;
5. Mathiowetz V, Volland G, Kashman N, Weber K. Adult norms for the Box and Block Test of manual dexterity. Am J Occup Ther. 1985 Jun;39(6):386–91.
6. Paternostro-Sluga T, Grim-Stieger M, Posch M, Schuhfried O, Vacariu G, Mittermaier C, et al. Reliability and validity of the Medical Research Council (MRC) scale and a modified scale for testing muscle strength in patients with radial palsy. J Rehabil Med. 2008 Aug;40(8):665–71.
7. Platz T, Vuadens P, Eickhof C, Arnold P, Van Kaick S, Heise K. REPAS, a summary rating scale for resistance to passive movement: item selection, reliability and validity. Disabil Rehabil. 2008;30(1):44–53.
8. Shinar D, Gross CR, Price TR, Banko M, Bolduc PL, Robinson RG. Screening for depression in stroke patients: the reliability and validity of the Center for Epidemiologic Studies Depression Scale. Stroke. 1986;17(2):241–5.
9. Ludwig K, Graf von der Schulenburg JM, Greiner W. German Value Set for the EQ-5D-5L. Pharmacoeconomics. 2018;1–12.
10. Buysse DJ, Reynolds CF, Monk TH, Berman SR, Kupfer DJ. The Pittsburgh sleep quality index: A new instrument for psychiatric practice and research. Psychiatry Res. 1989 Apr;28(2):193–213.
11. Shi D, Chen X, Li Z. Diagnostic test accuracy of the Montreal Cognitive Assessment in the detection of post-stroke cognitive impairment under different stages and cutoffs: a systematic review and meta-analysis. Neurol Sci. 2018 Apr 9;39(4):705–16.
12. Goul WR, Brown M. Effects of Age and Intelligence on Trail Making Test Performance and Validity. Percept Mot Skills. 1970 Feb;30(1):319–26.
13. Kernan WN, Ovbiagele B, Black HR, Bravata DM, Chimowitz MI, Ezekowitz MD, et al. Guidelines for the Prevention of Stroke in Patients With Stroke and Transient Ischemic Attack. 2014;2160–236.
14. Flöel A, Werner C, Grittner U, Hesse S, Jöbges M, Knauss J, et al. Physical fitness training in Subacute Stroke (PHYS-STROKE)--study protocol for a randomised controlled trial. Trials. 2014 Jan;15:45.
15. Dziura JD, Post LA, Zhao Q, Fu Z, Peduzzi P. Strategies for dealing with missing data in clinical trials: From design to analysis. Vol. 86, Yale Journal of Biology and Medicine. 2013.
16. Pedersen AB, Mikkelsen EM, Cronin-Fenton D, Kristensen NR, Pham TM, Pedersen L, et al. Missing data and multiple imputation in clinical epidemiological research. Clin Epidemiol. 2017;9:157–66.
17. Buuren S van, Groothuis-Oudshoorn K. mice : Multivariate Imputation by Chained Equations in R. J Stat Softw. 2011;45(3).
18. Little RJ, D'Agostino R, Cohen ML, Dickersin K, Emerson SS, Farrar JT, et al. The Prevention and Treatment of Missing Data in Clinical Trials. Vol. 367, New England Journal of Medicine. 2012. 1355-1360 p.
19. Ehrman JK, American College of Sports Medicine. ACSM's resource manual for Guidelines for exercise testing and prescription. Philadelphia [u.a.]: Wolters Kluwer Health/Lippincott Williams & Wilkins; 2010. 868 p.

20. Bateman A, Culpan FJ, Pickering AD, Powell JH, Scott OM, Greenwood RJ. The effect of aerobic training on rehabilitation outcomes after recent severe brain injury: A randomized controlled evaluation. Arch Phys Med Rehabil. 2001;82(2):174–82.

21. Sullivan TR, White IR, Salter AB, Ryan P, Lee KJ. Should multiple imputation be the method of choice for handling missing data in randomized trials? Stat Methods Med Res. 2018;27(9):2610–26.

22. Kwakkel G, Lannin NA, Borschmann K, English C, Ali M, Churilov L, et al. Standardized Measurement of Sensorimotor Recovery in Stroke Trials: Consensus-Based Core Recommendations from the Stroke Recovery and Rehabilitation Roundtable. Neurorehabil Neural Repair. 2017;31(9):784–92.
